# IMC10 and LMF1 mediate mitochondrial morphology through mitochondrion–pellicle contact sites in *Toxoplasma gondii*

**DOI:** 10.1242/jcs.260083

**Published:** 2022-11-15

**Authors:** Rodolpho Ornitz Oliveira Souza, Kylie N. Jacobs, Peter S. Back, Peter J. Bradley, Gustavo Arrizabalaga

**Affiliations:** ^1^ Department of Pharmacology and Toxicology, Indiana University School of Medicine, Indianapolis, IN 46202, USA; ^2^ Department of Microbiology and Immunology, Indiana University School of Medicine, Indianapolis, IN 46202, USA; ^3^ Molecular Biology Institute, University of California, Los Angeles, CA 90095, USA; ^4^ Department of Microbiology, Immunology, and Molecular Genetics, University of California, Los Angeles, CA 90095, USA

**Keywords:** *Toxoplasma gondii*, Mitochondrion, Membrane contact site, Inner membrane complex, LMF1

## Abstract

The single mitochondrion of *Toxoplasma gondii* is highly dynamic, being predominantly in a peripherally distributed lasso-shape in intracellular parasites and collapsed in extracellular parasites. The peripheral positioning of the mitochondrion is associated with apparent contacts between the mitochondrion membrane and the parasite pellicle. The outer mitochondrial membrane-associated protein LMF1 is critical for the correct positioning of the mitochondrion. Intracellular parasites lacking LMF1 fail to form the lasso-shaped mitochondrion. To identify other proteins that tether the mitochondrion of the parasite to the pellicle, we performed a yeast two-hybrid screen for LMF1 interactors. We identified 70 putative interactors localized in different cellular compartments, such as the apical end of the parasite, mitochondrial membrane and the inner membrane complex (IMC), including with the pellicle protein IMC10. Using protein–protein interaction assays, we confirmed the interaction of LMF1 with IMC10. Conditional knockdown of IMC10 does not affect parasite viability but severely affects mitochondrial morphology in intracellular parasites and mitochondrial distribution to the daughter cells during division. In effect, IMC10 knockdown phenocopies disruption of LMF1, suggesting that these two proteins define a novel membrane tether between the mitochondrion and the IMC in *Toxoplasma*.

This article has an associated First Person interview with the first author of the paper.

## Teaser

**Summary:** A tethering complex composed by LMF1 and IMC10 highlights the importance of pellicle–mitochondrion membrane contact sites in maintaining organelle morphology and distribution in *Toxoplasma gondii*.

## INTRODUCTION

*Toxoplasma gondii* is a highly successful intracellular pathogen that belongs to the phylum Apicomplexa (Black and Boothroyd, 2000) and is the causative agent of toxoplasmosis (Hill et al., 2005). This parasite can infect any nucleated cell in a plethora of homeothermic animals. It is estimated that ∼30% of the human population might be infected with *Toxoplasma* (Pappas et al., 2009). Although most infections are asymptomatic, toxoplasmosis is a severe problem for immunosuppressed patients (Porter and Sande, 1992) and in congenital infections (Khan and Khan, 2018). Drugs against these pathogens are limited, often toxic, and, for many, resistance is a serious challenge. Thus, the discovery of novel therapeutics is a priority.

A unique feature of this parasite is the presence of a single tubular mitochondrion, which is essential for parasite survival and a validated drug target. The mitochondrion of *Toxoplasma* is highly dynamic, showing different morphologies during the parasite propagation cycle and in response to stress factors (Charvat and Arrizabalaga, 2016; Jacobs et al., 2020; Ovciarikova et al., 2017). When the parasite is within a host cell, the mitochondrion is in a lasso shape, distributed along the periphery of the cell and adjacent to the pellicle. The pellicle in *Toxoplasma* is composed of the parasite plasma membrane and the inner membrane complex (IMC), which consists of a series of flattened membrane sacs and a supporting network of intermediate filaments. When in the extracellular environment, the mitochondrion collapses towards the apical end of the parasite. During this transition, some of the parasites can present an intermediate stage morphology called ‘sperm like’ (Ovciarikova et al., 2017). As soon as the parasite re-enters a cell, the mitochondrion recovers the lasso shape. It has been observed that when in the lasso shape, the mitochondrion has patches of its membrane in close proximity to the pellicle of the parasite, reminiscent of membrane contact sites (MCSs) (Ovciarikova et al., 2017).

Contact between both organelles is also observed during cell division. *Toxoplasma* divides by a specialized process called endodyogeny, where two daughter cells emerge within the mother cell (Hu et al., 2002). During this process, the IMC serves as a scaffold for the segregation and division of parasite organelles (Nishi et al., 2008). As there is only one mitochondrion per parasite, its division is tightly coordinated with the division of the rest of the parasite (Nishi et al., 2008; Verhoef et al., 2021). As the two nascent IMCs form during endodyogeny, the mitochondrion develops extensions along its length, which continue to grow as the daughter IMCs elongate. The branching mitochondrion is excluded from the daughter parasites until the latest stage of division, at which point mitochondrial branches enter the developing daughters moving along the IMC scaffold as the nascent parasites emerge from the mother cell (Nishi et al., 2008; Ovciarikova et al., 2017). Thus, the mitochondrion is highly dynamic as the parasite moves in and out of cells and during parasite division. Although the dynamics of the mitochondrion have been well described, our understanding of the mechanisms and the proteins that drive them remains vague.

Apicomplexan organisms must have evolved new ways to divide and distribute the mitochondrion, given the fact that most of the proteins involved in mitochondrial fission and fusion found in opisthokonta are not present in the genome of these organisms (Verhoef et al., 2021; Voleman and Dolezal, 2019). Recently our laboratory reported that a homolog of the yeast fission protein 1 (Fis1) is located at the outer mitochondrial membrane (OMM), but it is not essential for mitochondrial division or parasite survival *in vitro* (Jacobs et al., 2020). Interestingly, a dominant-negative version of this protein affects mitochondrial shape and positioning. While investigating the proteins that interact with Fis1, we found an alveolate-specific protein, TGGT1_265180, that localizes to the OMM. We have named this protein the Lasso maintenance factor 1 (LMF1) due to the remarkable phenotype observed in its absence. Parasites lacking LMF1 are not able to form a lasso-shaped mitochondrion. Instead, the organelle is either collapsed or sperm-like in intracellular parasites, suggesting that this protein is critical for mitochondrial shaping and positioning. In addition to the mitochondrial morphology phenotype, lack of LMF1 affects parasite fitness and mitochondrial segregation into daughter cells during division, which results in amitochondriate parasites and extracellular mitochondrial material (Jacobs et al., 2020).

LMF1 has no lipid binding or transmembrane domain, which opens questions about how this protein regulates mitochondrial shape. We previously determined that protein–protein interaction between LMF1 and Fis1 is required for the association of LMF1 with the mitochondrion and its function in maintaining the normal morphology of the mitochondrion. We hypothesize that LMF1 interacts with other proteins that facilitate the contact between the OMM and the parasite pellicle. In this work, we show that, indeed, LMF1 interacts with proteins located in the pellicle and the apical complex. Among these interactors, we found that the inner membrane protein IMC10 interacts with LMF1 to regulate mitochondrial shape and positioning. Inducible knockdown of IMC10 leads to loss of lasso shape and other mitochondrial abnormalities that phenocopy the effects of LMF1 deletion.

## RESULTS

### LMF1 interacts with proteins localized to different cell compartments

**Fig. 1.**
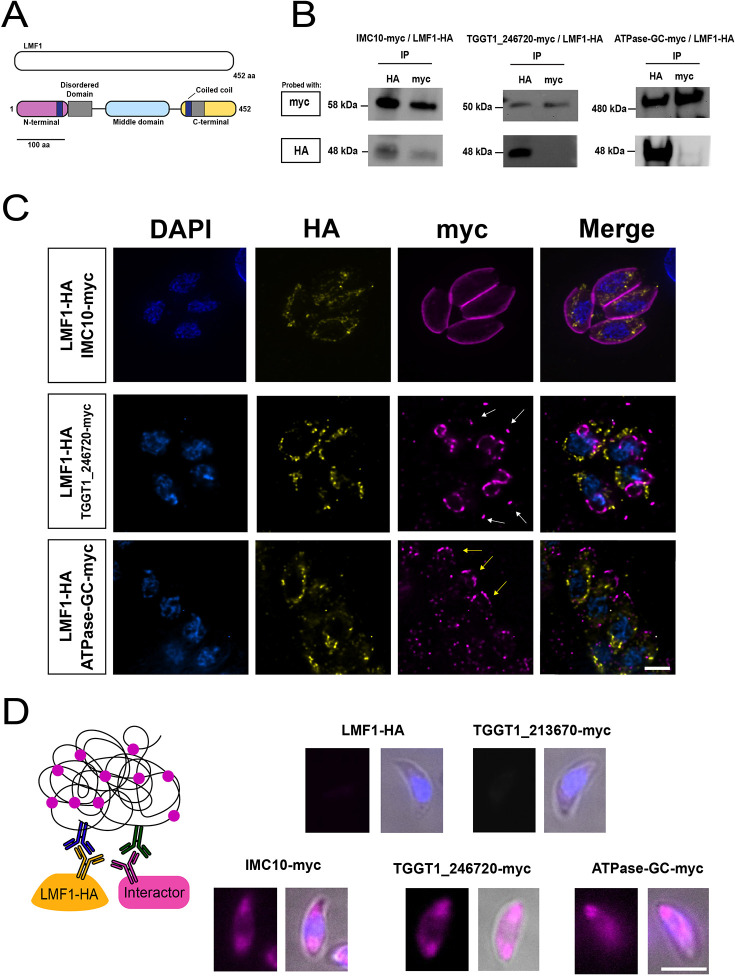
**Characterization of LMF1 interactors.** To investigate the localization of LMF1 interactors, we introduced sequences encoding an N-terminal Myc tag to the endogenous locus in the parasite strain expressing an HA-tagged LMF1. (A) Predicted domain architecture of LMF1. Schematic of LMF1 highlighting the three domains based on InterPro, Pfam and HHPred predictions. Magenta, N-terminal domain; cyan, middle domain; yellow, C-terminal domain; gray, predicted disordered domains; blue rectangles, predicted coiled-coil domains. (B) Reciprocal co-immunoprecipitation (IP) of putative LMF1 interactors was performed for the strains expressing LMF1–HA with IMC10–Myc, TGGT1_246720–Myc or ATPase-guanylyl cyclase (GC)–Myc. For each of the three dually tagged parasite strains, proteins were immunoprecipitated with either anti-HA or anti-Myc conjugated beads and probed with either Myc (for the interactor) or HA (for LMF1). (C) Intracellular parasites expressing the Myc-tagged versions of IMC10, TGGT1_246720 and ATPase-GC were stained for HA (yellow) and Myc (magenta). White arrows point at conoids, while yellow lines point at the location of the apical complex. (D) On the left, a schematic representation of the proximity ligation assay (PLA) approach is depicted. A signal is only expected when the two proteins labeled with the primary antibodies are in proximity of each other. Images show the result of PLA for the strain expressing only LMF1–HA and the dually tagged strains. TGGT1_213670 serves as a control as it was shown to not be an interactor of LMF1 by reciprocal IP (Fig. S1). Images were acquired using a Nikon 80i Eclipse. Results shown are representative of three repeats. Scale bars: 5 µm.

InterPro (https://www.ebi.ac.uk/interpro/search/sequence/) predictions for structured domains in the LMF1 sequence show that this protein might be organized in three different domains (N-terminal, middle and C-terminal domains) (Fig. 1A). InterPro predicts two intrinsically disordered domains [amino acids (aa) 104–181 and aa 319–376]. The presence of these domains was detected using MobiDB (https://mobidb.bio.unipd.it/). Intrinsically disordered proteins (IDP) or intrinsically disordered regions (IDR) are known for their lack of stable conformation in solution or their differential conformation upon binding to their partners (Zhou et al., 2019). HHPred (https://toolkit.tuebingen.mpg.de/tools/hhpred) predicts the presence of two internal coiled-coil (CC) domains (aa 51–89 and aa 282–312), one at the N-terminal and another one at the C-terminal. CC domains are very versatile domains present in proteins with different functions, such as cargo-binding proteins. However, it is also known that CC domains can serve as a scaffold for the assembly of supramacromolecular complexes (Truebestein and Leonard, 2016). The presence of these putative domains reinforces the hypothesis that LMF1 interacts with other proteins. Using a reciprocal BLAST querying of genomic sequences with the *Toxoplasma* LMF1 sequence, it was possible to recover 37 orthologs distributed into two phyletic groups – alveolates and cryptophytes. We identified homologs present in other coccidia (e.g. *Neospora*, *Sarcocystis* and *Hammondia*) and eimeriids, piroplasmids (*Babesia* spp. and *Cytauxzoon felis*) but not in *Cryptosporidium* and haemosporidians such as *Plasmodium* spp*.* (Fig. S1). Our phylogenetic analysis shows that LMF1 appeared very early in the cryptist heterotrophic algae *Guillardia theta* (26% identity with *Toxoplasma* LMF1). This organism possesses a very divergent sequence that has homology with LMF1. The chromerid *Chromera velia*, a phototrophic organism related to the apicomplexans, also encodes an LMF1 homolog that shares a 28% identity in amino acid composition with the *Toxoplasma* protein (Fig. S1).

To identify potential interactors of LMF1, we employed a yeast two-hybrid (Y2H) interaction screen. For this assay, we used full-length LMF1 as bait and analyzed over 95 million interactions using a *Toxoplasma* cDNA library. This screen yielded 257 positive clones, from which 69 putative interactors were identified (Table S1). These putative interactors were categorized based on the likelihood of interaction with LMF1 using the Predicted Biological Score (PrBS), which ranks interactors from A (highest confidence score) to D (lowest confidence score) (Table 1; Formstecher et al., 2005; Fromont-Racine et al., 1997). In total, there were four A interactors, six B, eight C, and 51 D. To narrow this list to those that are most likely to interact with LMF1, we considered their cellular localization. Given that LMF1 is localized to the outer mitochondrial membrane and that the mitochondrion has contact with the pellicle in intracellular parasites and the apical end in extracellular parasites, we narrowed our list to those proteins known to be localized to either the mitochondrion, the pellicle and the apical end of the parasite. The localization was based on a published spatial proteomics analysis (Barylyuk et al., 2020). This analysis resulted in a list of 15 proteins, with three located at the pellicle, seven apically and two mitochondrially (Table 1). A previous study used a CRISPR wide-genome screen to identify fitness-conferring genes in tissue culture grown *Toxoplasma* (Sidik et al., 2016). Interestingly, based on their fitness scores, most of the putative interactors are fitness-conferring during *in vitro* culture (Sidik et al., 2016).

**Table 1.**
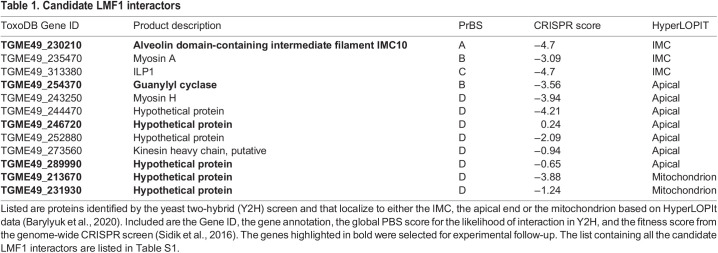
Candidate LMF1 interactors

The putative interactor with the highest confidence score was TGGT1_230210, also known as IMC10, which has been previously identified as a component of the IMC (Anderson-White et al., 2011). Interestingly, when we immunoprecipitated LMF1–HA, we identified IMC10 by mass spectrometry among the 18 identified proteins that had at least five peptides in the experimental immunoprecipitation and none in the control immunoprecipitation (Table S3). To further confirm the interaction with IMC10 and explore other proteins identified in the Y2H screen, we introduced a C-terminal Myc epitope tag to putative interactors in the cell line expressing the HA epitope-tagged LMF1 (LMF1–HA). For this analysis, we selected six proteins from the Y2H interactors list – IMC10, TGGT1_246720, ATPase-guanylyl cyclase (TGGT1_254370), TGGT1_213670, TGGT1_289990 and TGGT1_231930. TGGT1_246720 was selected because it had been previously identified as a putative interactor of the OMM protein Fis1, which also interacts with LMF1 (Jacobs et al., 2020). The ATPase-guanylyl cyclase, which has been shown to be localized to the apical complex of the parasite (Koreny et al., 2021; Long et al., 2017), was selected for analysis, given its potential regulatory role. TGGT1_289990 was selected because it is a predicted apical protein present only in coccidia (such as LMF1) and contains several disordered domains and a coiled-coiled domain, whereas TGGT1_213670 and TGGT1_231930 were selected due to their localization to the mitochondrion.

Once dual-tagged lines were established, we performed reciprocal immunoprecipitation assays using both HA- and Myc-conjugated magnetic beads (Fig. 1B). Using this technique, we determined that immunoprecipitation of LMF1 with HA beads brought down IMC10, TGGT1_246720, and ATPase-guanylyl cyclase, confirming these interactions. Similarly, we detected LMF1 when we immunoprecipitated IMC10, suggesting that the interaction between both proteins might be relatively stable (Fig. 1B). On the other hand, the interactions with TGGT1_289990, TGGT1_213670, and TGGT1_231930 could not be confirmed through immunoprecipitation of either LMF1 or the putative interactor (Fig. S2A).

Immunofluorescence assays (IFAs) of the dual-tagged lines confirmed the previously described localization of IMC10 to the IMC (Anderson-White et al., 2011) and ATPase-guanylyl cyclase to the apical end (Brown and Sibley, 2018) (Fig. 1C). TGGT1_246720 was previously determined to localize to the conoid (Koreny et al., 2021; Long et al., 2017), which we confirmed by IFA, but we also detected this protein at the budding daughter cells, in a similar pattern to that of the growing IMC (Fig. 1C). As for those that did not interact with LMF1 based on immunoprecipitation, TGGT1_213670 and TGGT1_231930 appeared to be in the mitochondrion, whereas TGGT1_289990 showed a punctate staining pattern throughout the parasite (Fig. S2B).

As a complementary confirmation of the interactions, we performed a proximity ligation assay (PLA) (Alam, 2018), which has been validated for use in *Toxoplasma* (Long et al., 2017; Mallo et al., 2021). We observed specific amplification of signal for all three interactors that had been confirmed by co-immunoprecipitation (Fig. 1D). Interestingly, the amplification of the signal did not corresponding to the shape of the mitochondrion, but the fluorescence was spread along the parasite. In contrast, when we applied PLA with TGGT1_213670, a protein that was determined not to interact with LMF1 based on co-immunoprecipitation, no signal amplification was detected. As an additional control, we used our parental LMF1–HA cell line with both antibodies, which, as expected, did not result in amplification. Together, these results show that IMC10, TGGT1_246720 and TGGT1_254370 appear to be true LMF1 interactors within the parasite.

### Ultrastructure expansion microscopy reveals the presence of LMF1 at contact sites

**Fig. 2.**
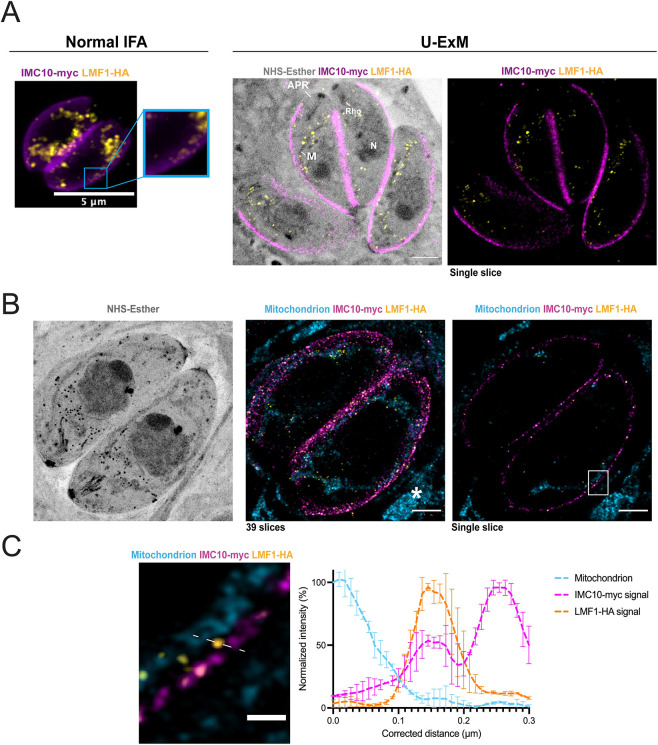
**Expansion microscopy shows colocalization between IMC10 and LMF1.** (A) Left, IFA of intracellular parasites stained with anti-HA (yellow) to detect LMF1 and anti-Myc (magenta) to detect the IMC. Box highlights a portion of the cell where the two signals are adjacent. Right, ultrastructure expansion microscopy (U-ExM) of intracellular parasites stained for LMF1–HA (yellow) and IMC10–Myc (magenta). On the right is the fluorescence signal showing the distribution of both proteins in the expanded parasites. On the left is an overlay of that image with the signal for NHS-ester, a total protein density marker. NHS staining allows for the visualization of structures such as the apical polar ring (APR), mitochondrion (M), rhoptries (Rho), and nucleus (N). (B) U-ExM panel of intracellular parasites. The left panel shows NHS staining. The center panel shows the *Z*-stacking of 39 slices of two parasites stained for the mitochondrion (cyan), LMF1–HA (yellow) and IMC10–Myc (magenta). Asterisks indicate the host cell mitochondrion. The right image is a single slice image. The white box highlights a region where the mitochondrion is in close contact with the IMC10 staining and LMF1. (C) Enlarged image of the boxed area showing the mitochondrion, LMF1 and IMC10 in close proximity to each other. The dashed line marks a region of proximity between signals, which was used to map fluorescence intensity for each signal. The graph shows the normalized fluorescence intensity (%) corresponding along the 0.3 µm line in the image on the left (real distance in the expanded image, ∼1.2 µm). Blue dotted line, mitochondrion signal; pink dotted line, IMC10 signal; yellow dotted line, LMF1 signal. The fluorescence intensity was calculated using ZEN Blue Software. Error bars indicate the s.d. of three independent measurements. See Movie 1 for the full *Z*-stack view. All images in this panel were acquired using both Zeiss LSM 800 (A panel) and 900 (B and C panel) with Airyscan processing. Results shown are representative of three repeats. Scale bars: 5 µm (A,B); 1 µm (C).

Using standard IFA and fluorescence microscopy, the staining pattern for LMF1 follows the length of the mitochondrion, as previously reported (Fig. 2A; Jacobs et al., 2020). In addition, we can detect patches of LMF1 in close proximity to the pellicle of the parasites (Fig. 2A, inset). With the advent of ultrastructure expansion microscopy (U-ExM), we revisited the localization of LMF1 to increase the level of detail and observe the distribution of the protein within the cell with higher resolution. Parasites expressing both LMF1–HA and IMC10–Myc were expanded in a water-expansible acrylate gel, and the gels were stained with anti-HA and anti-Myc antibodies. We also stained the gels with *N*-hydroxysuccinamide ester (NHS-ester), which binds to all primary amines of proteins and can reveal cell structures with a high level of detail (Dos Santos Pacheco et al., 2021). NHS staining allows us to visualize parasite structures such as the conoid, rhoptries and nucleus. Importantly, NHS also stains the mitochondrion, which allows us to track its shape without the use of an antibody (Fig. 2A). In expanded parasites, IMC10 is uniformly distributed along the periphery of the parasite (Fig. 2A, magenta), and LMF1 follows the shape of the mitochondrion (Fig. 2A, yellow and NHS-Esther). We also stained mitochondrion using an antibody against ATP synthase, which recognizes both the parasite and host mitochondrion, to confirm that the observed punctate signal for LMF1 is in the OMM (Fig. 2B). Using a single *z*-stack, it is possible to observe patches of the mitochondrion near the IMC10–Myc staining. Upon magnifying this region, we detect LMF1 dots in regions where the mitochondrion is near the pellicle, and in some of these contact regions, there is proximity between LMF1 and IMC10 staining (Fig. 2C). Drawing a line in an area where we see the mitochondrion, LMF1, and IMC10 near each other, we can determine the position of the peak intensity for each of the signals (Fig. 2C). The distance calculated was then converted using the expansion factor of each gel analyzed (our gels show an average of 4× expansion). This analysis shows that the peak intensity for LMF1 is near that of IMC10, suggesting proximity (Fig. 2C). Given the fact that LMF1 is near IMC10, in between the contact site zones between the mitochondrion and the pellicle, we hypothesize that LMF1 acts as a tether of this MCS by interacting with IMC10.

The pellicle is a detergent-resistant structure that can be isolated intact away from the rest of the parasite by using deoxycholate (DOC). Accordingly, we isolated the parasite pellicle using 1% DOC and analyzed it by IFA and western blotting to confirm the presence of LMF1. IFA of the isolated pellicles shows staining for IMC10 and, importantly, also for LMF1 (Fig. S3). Western blot analysis of the isolated pellicles reveals the presence of IMC10 and LMF1 in the pellicle fraction but not of the inner mitochondrial membrane-localized ATP synthase β-subunit (Fig. S3). These results using organelle extraction confirm the interaction of LMF1 with the pellicle, which is consistent with a role for LMF1 in mediating the contacts between the mitochondrion and the IMC.

### A decrease in the IMC10 protein levels does not affect *in vitro* propagation but disrupts mitochondrial morphology

**Fig. 3.**
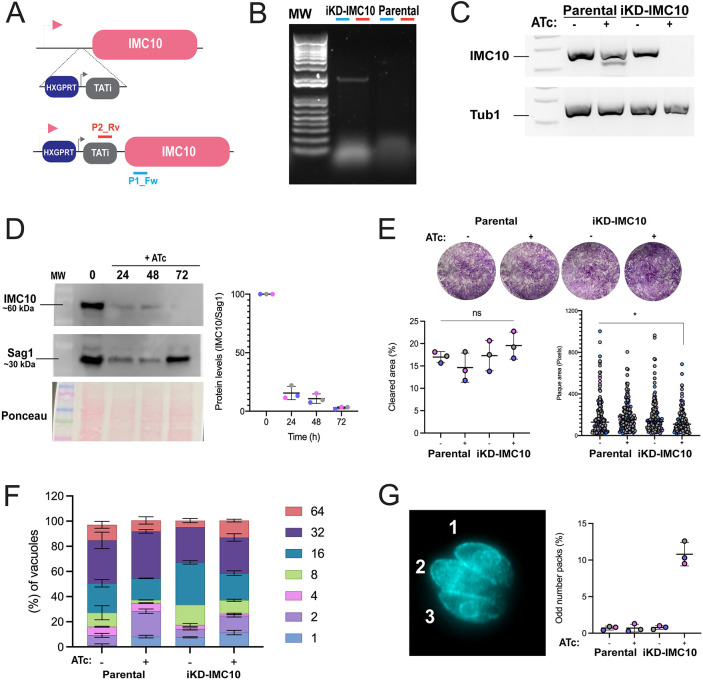
**Conditional knockdown of IMC10 does not affect parasite propagation *in vitro*.** (A) Schematic representation of replacement of the endogenous IMC10 promoter for the TATi promoter cassette, which allows for repression of IMC10 by addition of the tetracycline analog ATc. P2_Rv and P1_Fw indicate the positions of the primers used to confirm the promoter replacement. (B) PCR confirmation of promoter replacement using the primers depicted in A, which are expected to amplify a 2200 base pairs amplicon in the TATi strain but not the parental. Primers are represented in A. (C) Representative PCR using cDNA produced from parasites of the parental strain and the iKD-IMC10 strain grown with and without ATc for 24 h. PCR was done using specific primers for *IMC10* and tubulin, amplifying ∼150 bp for each gene. Results shown in B and C are representative of three repeats. (D) Western blot of iKD-IMC10 cell line treated for 24, 48 and 72 h with ATc. Blots were probed with anti-IMC10 and anti-Sag1 as a loading control. Representative of three independent experiments. On the right, quantification of protein levels by densitometry. Each replicate is represented in a different color. Error bars at means represent s.d. (*n*=3). The full membrane for this experiment is present in Fig. S6. (E) Representative plaque assay. The cleared and individual plaque area per well for either parental and knockdown cell lines grown with and without ATc after 5 days incubation period. Plaque assays were done in biological replicates (*n*=5), with error bars representing s.d. *P<0.05; ns, not significant (*P*>0.05) (unpaired, one-tailed *t*-test). (F) Doubling assay. Parasites were allowed to invade HFFs for 1 h, and the cultures were fixed after 48 h post-infection. The percentage of vacuoles containing 2, 4, 8, 16, 32, or 64 parasites was calculated for each condition. Doubling assays were performed in biological replicates (*n*=3). Error bars represent s.d. (G) Quantification of vacuoles showing an odd number of parasites. On the right is an example of a vacuole containing 03 parasites stained for acetylated tubulin. Counts were extracted from the doubling assays. Error bars represent s.d., *n*=3.

Based on a whole-genome CRISPR selection, IMC10 is predicted to be fitness conferring for tachyzoites in tissue culture (fitness score −4.01) and likely to be essential (Sidik et al., 2018). Accordingly, we generated a conditional knockdown (iKD) strain by replacing the endogenous *IMC10* promoter with a tetracycline repressible one (Fig. 3A). We confirmed the insertion by PCR (Fig. 3B) and showed that the addition of the tetracycline analog anhydrotetracycline (ATc) results in a substantial reduction of *IMC10* mRNA levels in the iKD-IMC10 but not in the parental strain (Fig. 3C). To test whether the IMC10 protein levels were also affected, we exposed parasites to ATc for 24 h, 48 h and 72 h and collected total protein extracts from intracellular parasites for western blot analysis. We saw a substantial decrease in the IMC10 protein levels after 24 h of treatment (∼85% decrease), and after 72 h, we observed a 97% decrease in the IMC10 protein levels (Fig. 3D).

To assess whether the knockdown would interfere with the replication of these parasites, we performed a plaque assay in which the parasites were incubated for 5 days with or without ATc. The plaque area cleared after 5 days was similar among the conditions and strains tested. When the individual plaque area was measured, a slight decrease in the plaque area could be observed in the iKD-IMC10 upon ATc treatment (Fig. 3E). Together, this data shows that in the presence of substantially lower levels of IMC10, the parasites are still able to complete a full intracellular cycle. To confirm the lack of a growth phenotype, we performed a doubling assay with the iKD-IMC10 and parental strains in the presence and absence of ATc. After 48 h, we counted the number of parasites within the vacuoles and tabulated the percentage of vacuoles with a specific number of parasites across strains and conditions. As indicated by the plaque assay, no proliferation phenotype was observed, with all conditions having the same distribution of vacuole sizes (Fig. 3F). Normally, during endodyogeny, two daughter cells form within a mother parasite. Nonetheless, events with more than two daughter cell budding can occur, although this occurs very rarely for wild-type parasites (Hu et al., 2002). Interestingly, among the vacuoles counted, we could observe the presence of many containing an abnormal number of parasites. In our doubling assay counts, we could observe the appearance of vacuoles containing an abnormal number of parasites. In the presence of ATc, 10.8±1.609% (mean±s.d.) of vacuoles contained an abnormal number of parasites, which is a significantly higher proportion than the ∼0.7±0.2% observed with untreated parasites (Fig. 3G). In sum, these results indicate that, given the significant reduction in protein levels in our knockdown strain, IMC10 is unlikely to be essential, contrary to what was suggested by the low fitness score.

**Fig. 4.**
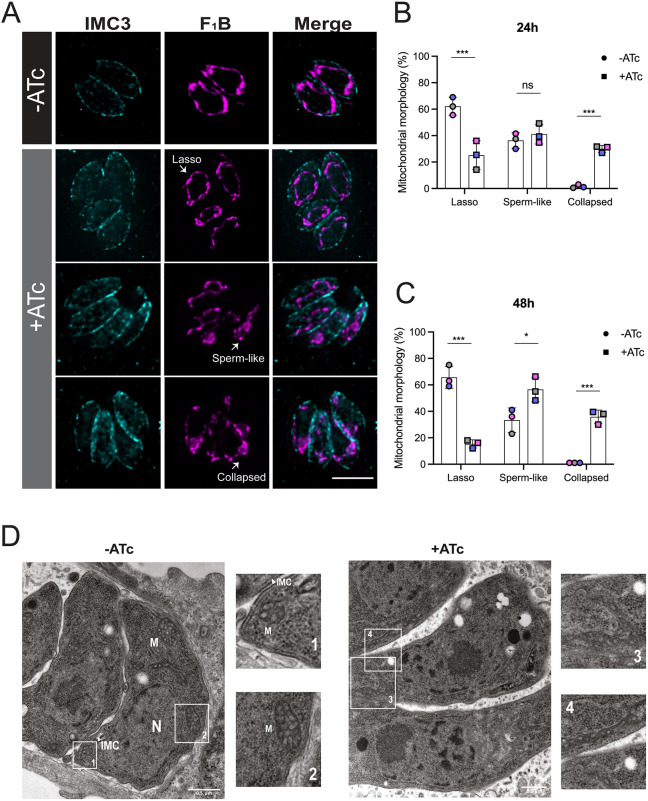
**IMC10 knockdown disrupts mitochondrial morphology.** (A) Intracellular parasites of the iKD-IMC10 strain were grown without (–) or with (+) ATc to regulate IMC10 expression. Parasites were stained for IMC3 (cyan) and F_1_B-ATPase (magenta). Images were taken 24 h post treatment. Scale bar: 5 µm. (B,C) Percentage of parasites with each of the three different morphologies for parasites grown in the absence and presence of ATc after 24 (B) and 48 h (C). Data are mean±s.d. of three replicates; at least 150 non-dividing vacuoles with intact IMC per sample were counted. ***P<0.001; *P<0.05; ns, not significant (*P*>0.05) (one-way ANOVA with Tukey post-correction). (D) Representative transmission electron microscopy (TEM) images of induced and non-induced cell lines 24 h post infection. Insets 1 to 4 show detailed structures, including the inner membrane complex (IMC), the nucleus (N), and the mitochondrion (M). IFA images were acquired using a Nikon 80i Eclipse. Images shown in D are representative of a single repeat. Scale bars: 500 nm.

Given the interaction between LMF1 and IMC10 and the role of LMF1 in mitochondrial morphology, we determined whether the lack of IMC10 affected normal mitochondrial dynamics. Normally, intracellular parasites present mostly lasso-shaped mitochondrion, whereas extracellular parasites present both sperm-like and collapsed mitochondrion (Jacobs et al., 2020; Ovciarikova et al., 2017). Lack of LMF1 leads to most intracellular parasites having either a sperm-like or collapsed mitochondrion (Jacobs et al., 2020). Accordingly, we monitored the morphology of mitochondrion in iKD-IMC10 parasites grown in the presence and absence of ATc (Fig. 4A). In addition, to focus on non-dividing parasites with an intact IMC, we also stained for IMC3 (Fig. 6A) (Gubbels et al., 2004). After 24 h post infection, parasites without ATc showed a higher percentage of lasso (62.16±6.75%; mean±s.d.) in comparison to parasites in the presence of ATc (25.23±10.86%) (Fig. 4B). In addition, parasites under ATc treatment showed an increase in the proportion with a sperm-like shape (41.1±7.37% versus 36.33±4.77% for parental) and a collapsed mitochondrion (30.01±2.58% versus 1.5±1.08% for parental). This phenotype was also observed after 48 h infection in the presence of ATc, with only 14%±2.01 of parasites showing a lasso mitochondrion in contrast to 61.01±2% of parental (Fig. 4C). To confirm the phenotype we observed by IFA at a higher resolution, we looked at the ultrastructure of the parasite by electron microscopy (EM). The EM images show clear differences in mitochondrial positioning after IMC10 ablation (Fig. 4D). In non-treated cells, it is possible to visualize distinct patches of the mitochondrion in contact with the IMC (Fig. 4D, boxes 1 and 2). The sections of mitochondrion observed in non-treated parasites show the expected tubular shape. Upon IMC10 knockdown, most parasites exhibit a sperm-like mitochondrion and/or a collapsed mitochondrion with large bundles of mitochondrion accumulated in various regions of the parasite (Fig. 4D, boxes 3 and 4).

**Fig. 5.**
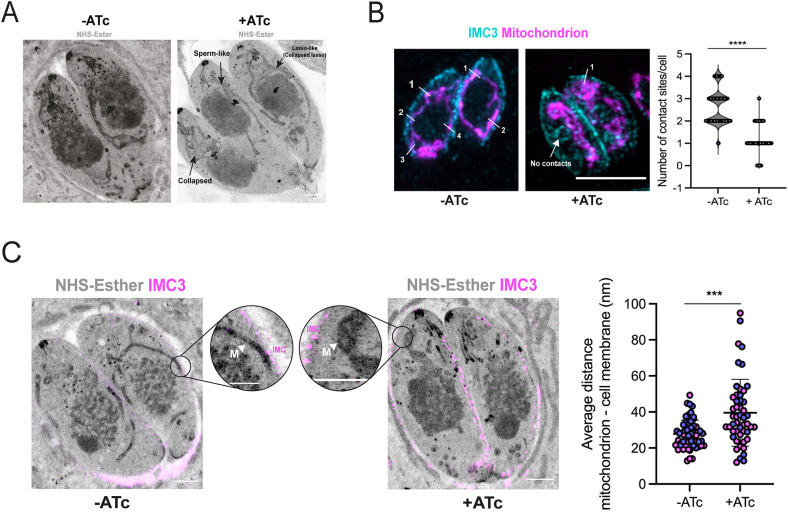
**iKD-IMC10 cell lines show defects in mitochondrion position.** (A) Representative figure of parasites in the presence or absence (control) of ATc visualized by ExM. Parasites were expanded and stained with NHS-ester (protein density marker) to highlight cellular structures. The three main mitochondrial phenotypes observed in the iKD-IMC10 in the presence of ATc are highlighted. (B) The mean±s.d. number of visible IMC–mitochondrion contacts. Vacuoles containing a maximum of 4 parasites were counted, a total of 50 parasites per replicate (*n*=3). ****P<0.0001 (one-way ANOVA with Tukey post-correction). (C) Average distance from the pellicle (represented by the IMC3 staining in magenta) to the mitochondrion (NHS staining) was calculated in the expanded parasites by measuring the distance between both organelles at their closest point (arrowhead indicates the mitochondrion). A total of 30 parasites were counted in two biological replicates (*n*=2). ***P<0.001 (one-way ANOVA with Tukey post-correction). Error bars represent s.d. Each replicate is represented by a different color. All images in the panels were acquired using a Zeiss LSM800 microscope with Airyscan processing and were taken 24 h post treatment. Scale bars: 5 µm.

To quantify this phenotype, we used U-ExM and NHS-ester staining to analyze the cell structure of parasites lacking IMC10 (Fig. 5A). As with IFA, ExM reveals clear disruption of mitochondrial morphology when the iKD-IMC10 parasites are exposed to ATc. Fig. 5A shows a vacuole of the knockdown strain grown in ATc, showing all three morphologies: lasso, sperm-like and collapsed (Fig. 5A). Interestingly, we note that although the mitochondrion appears lasso-like in some parasites, it is not continuous, and exhibits breaks along its length (Fig. 5A).

Our results confirm that IMC10 and LMF1 are acting as a tether for the mitochondrion–pellicle contact sites, and the decrease of IMC10 expression can lead to altered mitochondrial morphology. To check whether the IMC10 knockdown is affecting mitochondrion–pellicle MCSs, we counted in how many points the mitochondrion is touching the IMC. For that experiment, we treated parasites for 48 h with ATc (or without as a control) and performed IFA, staining for mitochondrion (F1B-ATPase) and the IMC (IMC3) (Fig. 5B). We quantified 50 parasites per condition (*n*=3) using the same criteria we used for the mitochondrion morphology counts – non-dividing parasites, packs of two and four parasites, and an intact IMC. Non-treated parasites showed a higher number of mitochondrion–pellicle contact zones (2.63±0.754 contacts per cell; mean±s.d.) in comparison to the treated parasites (1.146±0.595 contacts per cell) (Fig. 5B). As the expansion per se does not alter the mitochondrial morphology or its positioning, it was possible to observe regions of apposition of the mitochondrion and the parasite pellicle, and to measure the distance between the two structures. To quantify this observation, we selected 30 parasites (*n*=2), and based on the number of contact sites observed in the treated cells (∼1 contact per cell), we decided to measure one point of distance using, as a reference, the staining for IMC3 and the NHS staining for the mitochondrion. As previously reported, we converted the expanded distance by the average expansion factor of the gels (4×). Based on our measurements, untreated parasites showed a distancing of 24.67±7.78 nm (mean±s.d.), which is close to that previously described (26.23±12.02 nm; Ovciarikova et al., 2017). There was a significant shift in the distance from the mitochondrion to the pellicle in the treated knockdown strain, with an average of 36.7±16.6 nm (Fig. 5C).

It is interesting to note that the sperm-like mitochondrion is sometimes accumulated very close to the IMC, although it is not possible to know if this is the result of active tethering or if it is just a random effect. Through U-ExM, we can observe that the sperm-like mitochondrion has extended along the cell body towards the apical end of the parasite. In addition, parasites do not show any significant structural differences in the apicoplast and endoplasmic reticulum (ER) upon IMC10 knockdown (Fig. S4). Together, these results suggest that the presence of IMC10 is critical for the mitochondrial morphology in intracellular parasites and likely plays a role in tethering the mitochondrion to the pellicle.

### IMC10 iKD affects cell division and mitochondrial inheritance

**Fig. 6.**
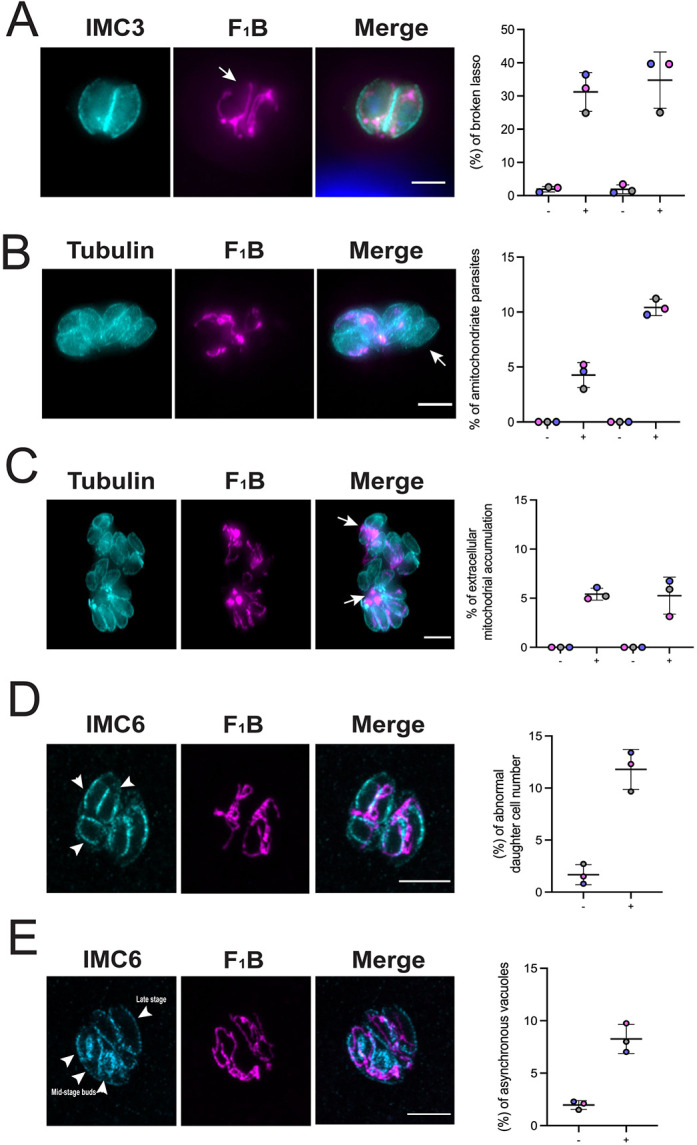
**IMC10 knockdown exhibit other mitochondrial distribution and division phenotypes.** IFA of knockdown parasites stained for IMC3 or acetylated tubulin (cyan) and F_1_B-ATPase (magenta) showed aberrant phenotypes. (A) Broken lasso. (B) Amitochondriate parasites. (C) Accumulation of mitochondrion material outside of the cells within the same vacuole. (D,E) IFA of knockdown parasites stained for IMC6 (cyan) and F_1_B-ATPase (magenta) showing aberrant phenotypes. Arrows in A–C highlight, respectively, a broken mitochondrion, a amitochondriate parasite and mitochondrial material accumulated outside of the parasites. For A, B, and C, images were acquired 24 h post treatment. In the graph, the left two columns are 24 h post treatment, and right 48 h post treatment. −, without ATc, + with ATc. (D) Aberrant number of budding cells within the same mother. Arrowheads indicate three daughter cells budding inside of a single mother. (E) Asynchronous vacuoles in which parasites are in different stages of division. Arrowheads indicate two different phenotypes within the same vacuole, abnormal daughter cell number and lack of synchronicity. For both D and E, parasites were inspected after 48 h in the presence of ATc. All graphs represent the mean±s.d. percentage of vacuoles with the related phenotype. At least 150 vacuoles per sample were inspected. For all graphs, *n*=3. Each replicate is represented by a different color. Images were acquired using a Nikon 80i Eclipse microscope. Scale bars: 5 µm.

As noted above, the lasso-shaped mitochondrion in the absence of IMC10, although being present in some parasites, did not appear contiguous as normal (Fig. 6A, arrow). Given that observation, we counted 150 vacuoles where the mitochondrion was in lasso shape in the parasites with and without ATc treatment and assessed for the broken lasso phenotype. In knockdown parasites grown without ATc, only 2.34±0.56% (mean±s.d.) of parasites exhibited a broken lasso. By contrast, after 24 h in ATc, the percentage of parasites with a broken lasso-shaped mitochondrion increased to 32.3±6.5% (Fig. 6A). We observe the same phenotype after 48 h in ATc (Fig. 6A).

During inspections of IFA images of parasites lacking IMC10, we detected numerous other phenotypes, probably related to defects during cell division upon IMC10 knockdown. Specifically, we detected parasites without a mitochondrion (amitochondriate) (Fig. 6B) and accumulation of mitochondrial material outside of the parasites (Fig. 6C, arrowhead). All these phenotypes were quantitated, and we observed substantial differences between the parasites grown without and with ATc for either 24 or 48 h (Fig. 6A–C). Interestingly, all these phenotypes were also observed in the LMF1-knockout parasite strain (Jacobs et al., 2020).

As we observed the appearance of an abnormal number of parasites within the same vacuole, we decided to look whether this phenotype is related to a defect during the division process. To monitor daughter cells, we stained parasites grown with and without ATc with IMC6. We considered any parasite with either one or more than two daughter cells as undergoing abnormal division. We observed that after 48 h in the presence of ATc, the number of cells showing abnormal division was 12.3±0.8% (mean±s.d.) within the population, which is substantially higher than the 1.5±0.8% observed in the absence of ATc (Fig. 6D). Another characteristic observed upon IMC10 ablation was that parasites within the same vacuole were dividing asynchronously. Among all vacuoles with dividing parasites in the ATc-grown parasites, 9.7±2% had parasites in different time points of division. By contrast, only 2.1±0.32% of those without ATc treatment exhibited this phenotype (Fig. 6E). This result points out that IMC10 is important for cell division, even though the protein is not essential for the lytic cycle of the parasite.

### IMC10–LMF1 interaction promotes mitochondrial distribution during endodyogeny

**Fig. 7.**
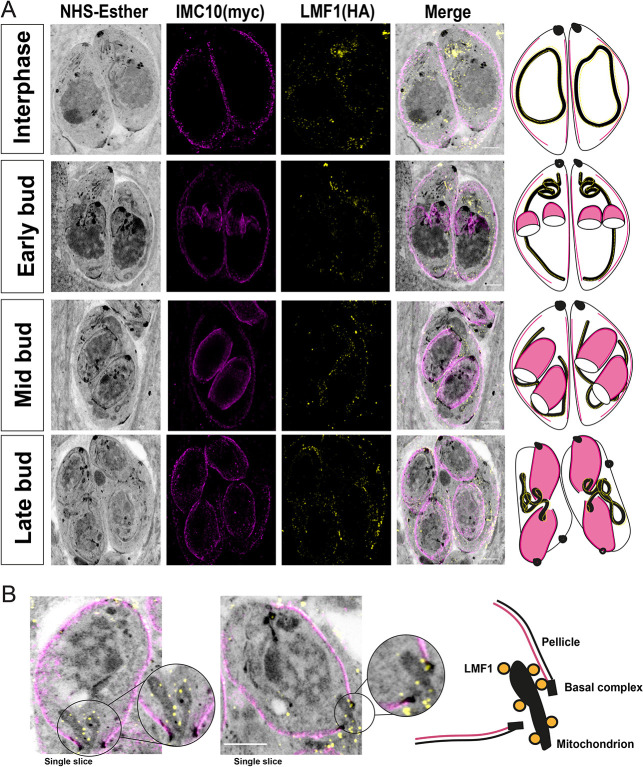
**LMF1 and IMC10 interact during mitochondrial distribution.** (A) U-ExM of intracellular parasites stained for LMF1-HA (yellow) and IMC10-Myc (magenta). On the left is the fluorescence signal showing the distribution of both proteins in the expanded parasites, followed by an overlay of that image with the signal for NHS-ester. On the right is a representation of the observed pattern of localization. See Movies 2–5 for the full *Z*-stack view. (B) Left, detail of two late-stage division cells with an emerging mitochondrion branch. Right, scheme depicting the relative localization of the proteins, including structures such as the basal complex, mitochondrion, LMF1 (yellow circles), and pellicle. Images were acquired using a Zeiss LSM 800 microscope with Airyscan processing. Scale bars: 5 μm. Results shown are representative of three repeats.

Based on our results, IMC10 is important for mitochondrial distribution among daughter cells during division. As was mentioned above, mitochondrial distribution is also affected in cells lacking LMF1 (Jacobs et al., 2020). Accordingly, we examined the localization dynamics of these proteins during mitochondrial inheritance during cell division using U-ExM. We imaged the dual-tagged LMF1–HA IMC10–Myc cell line at different time points of cell division and followed the distribution of the mitochondrion based on the NHS staining (Fig. 7A). It was previously observed that the mitochondrion is one of the last organelles to enter the daughter cells during endodyogeny (Nishi et al., 2008). During interphase, we can observe parasites presenting a full lasso, which appears to have contact with the pellicle. As cell division progress, the lasso-shaped mitochondrion is opened, and it starts to move along the mother cell during early and mid-budding. Interestingly, the basal body of these stages is larger, and it is not possible to see any mitochondrion inside the daughter cells. In late budding, it is possible to observe that as the daughter cells grow, the basal complex tightens, and the mitochondrion branches start entering the daughter cells. The mitochondrion branches appear to be in close proximity to the pellicles of the daughter parasites (Fig. 7A,B; for images of non-expanded parasites, see Fig. S5.). At the same time, it is possible to observe that the proximity between LMF1 and IMC10 occurs during mitochondrion inheritance (Fig. 7B). Together, these results suggest that the membrane contact between the mitochondrion and the pellicle in *Toxoplasma* happens during cell division, and it is important for organelle division and distribution to the daughter cells, explaining the mitochondrial distribution phenotypes observed with a knockout of either LMF1 or IMC10.

## DISCUSSION

Membrane contact sites (MCSs) are defined as regions where membranes from two compartments are tethered in close apposition (∼30 nm) and in which specific proteins and/or lipids are enriched (Eisenberg-Bord et al., 2016; Prinz, 2014). These contact sites are important for several physiological processes, including ion (Raturi et al., 2016) and lipid exchange (Aaltonen et al., 2022). The importance of interorganellar contact sites also extends to evolutionarily early branching organisms (Ovciarikova et al., 2022). In *Toxoplasma*, contact between organelles has been described between the mitochondrion and the apicoplast (Nishi et al., 2008), the ER (Mallo et al., 2021) and the IMC (Jacobs et al., 2020; Ovciarikova et al., 2017), and between the ER and the apicoplast (Tomova et al., 2009). An apicoplast-localized two-pore channel (TPC) has been determined to be responsible for mediating apicoplast–ER contact sites and Ca^2+^ exchange between these organelles (Li et al., 2021). Nonetheless, the function and components of other contact sites, including those between the mitochondrion and the pellicle, are not well understood. We previously described the identification and characterization of an alveolate-specific protein, LMF1, that localizes at the mitochondrion outer membrane, and that is essential for positioning the mitochondrion to the periphery of the parasite (Jacobs et al., 2020). In the absence of LMF1, the mitochondrion loses its typical lasso shape in intracellular parasites and collapses to one of either of the ends of the parasite. Accordingly, we hypothesized that LMF1 is part of a tethering complex that links the OMM to the parasite pellicle.

In this study, we focused on the proteins that collaborate with LMF1 in mitochondrion shaping and distribution. We confirmed three putative interactors first identified through a Y2H screen – IMC10, a hypothetical protein (TGGT1_246720), and ATPase-guanylyl cyclase. TGGT1_246720 is present in the conoid of the mother cell, but it is also present in what appears to be the IMC of the daughter cells. ATPase-guanylyl cyclase is an integral membrane protein localized towards the apical end of the parasite that is involved in Ca^2+^ and phosphatidic acid (PA) signaling during egress, motility and microneme secretion (Bisio et al., 2019; Brown and Sibley, 2018; Yang et al., 2019b). Although TGGT1_246720 and ATPase-guanylyl cyclase have been previously characterized, those studies did not investigate the shape of the mitochondrion in their absence. Given the rapid transition of the mitochondrion from a lasso to a collapsed morphology as the parasites exit the host cell, it is plausible that ATPase-guanylyl cyclase and other signaling proteins that regulate egress are involved in regulating mitochondrial morphology. Similarly, the presence of TGGT1_246720 in daughter cells could suggest that its interaction with LMF1 is related to mitochondrial inheritance. Further work is needed to understand the role of these two proteins in the mitochondrial dynamics in *Toxoplasma*.

Owing to the likely contact between the OMM, where LMF1 is localized, and the parasite pellicle, we were particularly intrigued by those interactors that are known to be part of the IMC. The inner membrane complex is part of the parasite pellicle, and it is composed of flattened sacs termed alveoli, supported by the subpellicular network (Mann and Beckers, 2001) on the cytoplasmic face and interacts with the microtubule cytoskeleton of the parasite (reviewed by Harding and Meissner, 2014; Harding et al., 2019). Attempts to tag ILP1 in the LMF1–HA-expressing strain failed, so we could not confirm the interaction. Nonetheless, disruption of ILP1 has been reported to affect the mitochondrion (Chen et al., 2015), although, given the pleiotropic effects of ILP1 knockdown, it is unclear whether this effect is specific. Nonetheless, we were able to confirm the interaction between LMF1 and IMC10 with various complementary approaches. IMC10 is an alveolin-containing protein localized to the pellicle (Anderson-White et al., 2011), and its expression is upregulated during cell division (Behnke et al., 2010). Surprisingly, the knockdown of IMC10 did not affect fitness, suggesting that it is not essential for propagation in tissue culture. This contrasts with what would have been expected based on the negative fitness score from the CRISPR high-throughput screen (Sidik et al., 2016). It is possible that under our knockdown conditions, some protein is still expressed, which is enough to maintain normal propagation. Interestingly, other IMC proteins with the same expression pattern as IMC10, such as IMC14 and IMC15, have also been shown not to be essential for parasite propagation in culture (Dubey et al., 2017). Regardless, knockdown of IMC10 significantly affected the mitochondrial morphology in intracellular parasites, phenocopying the effects of knocking out LMF1. It is noticeable that the growth phenotype presented by the LMF1-knockout cell lines is stronger than our iKD-IMC10 (Jacobs et al., 2020). We hypothesize that this might be due to a more critical role for LMF1 in mitochondrial dynamics and its interaction with other proteins involved in this process, including those that ensure the correct inheritance of the organelle. The fact that the lack of either LMF1 or IMC10 results in the same phenotypes, and that we confirmed their interaction with three different approaches, strongly suggests that these two proteins are part of an alveolate-specific tethering complex. A study recently reported that porin mediates the contact between the mitochondrion and the ER in these parasites; knockdown of a mitochondrial porin led to morphological defects in both the mitochondrion and the ER (Mallo et al., 2021). Thus, it is evident that contact and tethering to other structures of the parasite are central to maintaining the morphology of the mitochondrion.

In yeast, a protein called Num1 is the tether that mediates mitochondrial–cortex contact sites and confers proper mitochondrial segregation during cell division (Kraft and Lackner, 2017). This protein is structurally composed of internal EF-hands, a coiled-coiled domain that binds to the mitochondria, and a pleckstrin homology domain (PH domain) that binds to the plasma membrane lipids (Ping et al., 2016). Computational analysis performed by us shows that LMF1 has no lipid-binding domains, which reinforces the idea that protein–protein interactions mediate *Toxoplasma*'s mitochondrial dynamics. Our previous studies suggest that LMF1 associates with the OMM through an interaction between its C-terminal domain and Fis1 (Jacobs et al., 2020). In the LMF1 Y2H, the interaction region between IMC10 and LMF1 was a part of the IMC10 C-terminal, probably connecting to the N-terminal of LMF1. More studies are necessary to describe the minimal region that determines the interactions among these proteins. Regulation of mitochondrial division in related apicomplexans such as *Plasmodium* is still an open question. Recently it was reported that a Fis1 homolog in *Plasmodium* is dispensable for mitochondrial division. As knockout of *Plasmodium falciparum* (Pf)Fis1 showed no effect in either parasite growth or mitochondrial division, the authors hypothesized that other proteins participate in this process (Maruthi et al., 2020). Interestingly, *Plasmodium* does not seem to encode an LMF1 homolog.

IMC10 knockdown affected not only mitochondrial morphology but also cell division. After induction, it was possible to observe asynchronous cell division, leading to an increase in the number of polyploid cells and vacuoles with an abnormal number of parasites. These division defects might also relate to the defects in mitochondrial distribution to the daughter cells, which result in amitochondriate parasites and excess extracellular mitochondrial material within the vacuoles. Importantly, the lack of LMF1 also results in mitochondrial inheritance defects, suggesting that the LMF1–IMC10 complex plays a role during endodyogeny.

Mitochondrial division in *Toxoplasma* is a tightly regulated process within daughter cell budding (Nishi et al., 2008). The organelle is one of the last to enter the newly formed cells, and it is possible to observe branches of this organelle emerging and entering daughter cells in the late endodyogeny stages (Nishi et al., 2008; Verhoef et al., 2021). Using U-ExM, we could observe what appear to be LMF1–IMC10 complexes upon mitochondrial distribution to the daughter cells. This data strongly suggests that the formation of MCSs is important for proper mitochondrial inheritance, given the fact that disturbing both IMC10 and LMF1 causes mitochondrial segregation defects and excessive accumulation of mitochondrial material in the residual body. The understanding of the proteins involved in mitochondrial division in apicomplexan parasites is still very limited (Verhoef et al., 2021; Voleman and Dolezal, 2019). Apicomplexan parasites do not appear to encode homologs of bacterial FtsZ and instead encode a set of dynamin-related proteins (Drp) (Morano and Dvorin, 2021). In *Toxoplasma*, DrpA is involved in apicoplast division (van Dooren et al., 2009), DrpB in secretory organelle biogenesis (Breinich et al., 2009), and DrpC plays a role in vesicle transport (Heredero-Bermejo et al., 2019) and mitochondrial fission (Melatti et al., 2019), although the association of this latter protein with the mitochondrion is still unclear. Indeed, DrpC is intriguing because this protein lacks its GTPase effector domain (GED). The fact that there is not a direct involvement of a canonical dynamin-related protein and that finding that the absence of Fis1 does not affect mitochondrial morphology in this parasite leads to the question of what the other proteins mediate mitochondrial fission and inheritance in the parasite. Our identification of a novel and unique tethering complex that mediates mitochondrial contact with the pellicle provides a handle with which to study the morphodynamics of the mitochondrion of this important pathogenic parasite. Future studies of other components of this tethering complex, especially those involved in its regulation, will shed light on this important aspect of biology of *Toxoplasma* and potentially reveal novel avenues for therapeutic interventions.

## MATERIALS AND METHODS

### Parasite culture and reagents

All the parasite strains were maintained via continued passage through human foreskin fibroblasts (HFFs) purchased from ATCC (SCRC-1041) and cultured in Dulbecco's modified Eagle's medium (DMEM) high glucose, supplemented with 10% fetal calf serum (FCS), two mM L-glutamine, and 100 U penicillin/100 µg streptomycin per ml. The cultures were maintained at 37°C and 5% CO_2_. Parasites used in this study were of the strain RH lacking hypoxanthine-xanthine-guanine phosphoribosyl transferase (HPT) and Ku80 (RHΔHPTΔku80) (Huynh and Carruthers, 2009). Cells were inspected for mycoplasma contamination using a Venor^TM^ GeM Mycoplasma Detection Kit PCR-based (Sigma, MP0025-1KT).

### Phylogeny and domain prediction

Domain prediction was determined by the InterPro 87.0 (https://www.ebi.ac.uk/interpro/search/sequence/) tool using the full LMF1 amino acid sequence. To confirm the prediction of intrinsically disordered domains, we used the MobiDB (https://mobidb.bio.unipd.it) tool (Piovesan et al., 2021). In order to uncover the presence of other significant domains, we used HHPred. Coiled-coil domains were predicted using the Quick2D toolkit on HHPred (https://toolkit.tuebingen.mpg.de/tools/hhpred). Phylogeny was performed using the tBLASTp tool (https://blast.ncbi.nlm.nih.gov/Blast.cgi?PAGE=Proteins) to compare amino acid sequences against the LMF1 protein. Homologs among apicomplexans and other organisms were confirmed by searches using the ToxoDB blast tool (https://toxodb.org/toxo/app; Amos et al., 2022). For the phylogenetic tree, we used the OrthoMCL DB tool (https://orthomcl.org/orthomcl/app/; Zdobnov et al., 2021). LMF1 ortholog group (OG6_176398) was used to detect homologs and determine their phyletic distribution. A cut-off of 1×10^−5^ was used in this search. As a second approach, we used the LMF1 protein sequence as bait to look for homologs within the vEuPathDB database (https://veupathdb.org/veupathdb/app/; Amos et al., 2022). In total, 37 sequences were found for this phyletic group, with an average of 57.6% identity among all sequences. Accessions for the sequences used in this work are: *Sarcocystis neurona* N3 (sneu|SN3_01200745), *Cystoisospora suis* strain Wien I (csui|CSUI_005550), *Besnoitia besnoiti* strain Bb-Ger1 (bbes|BESB_048460), *Neospora caninum* Liverpool (ncan|NCLIV_040070), *Toxoplasma gondii* GT1 (tggt|TGGT1_265180), *Toxoplasma gondii* ME49 (tgon|TGME49_265180), *Eimeria tenella* Houghton 2021 (etht|ETH2_1406700), *Cyclospora cayetanensis* strain CHN_HEN01 (ccay|cyc_05565), *Guillardia theta* (strain CCMP2712) (Cryptophyte) (gthe|L1IU32) and *Chromera velia* CCMP2878 (cvel|Cvel_23028).

### Yeast two-hybrid

Yeast two-hybrid (Y2H) screening was performed by Hybrigenics Services, S.A.S., Paris, France. The coding sequence for LMF1 (aa 1–452, XP_002368647.1) was PCR amplified and cloned into a pB66 as a C-terminal fusion with the Gal4 DNA-binding domain (Fromont-Racine et al., 1997). 46 million clones (5-fold the complexity of the library) were screened using a mating approach with YHGX13 (Y187 *ade2-101::loxP-kanMX-loxP, matα*) and CG1945 (*matα*) yeast strains as previously described (Fromont-Racine et al., 1997). 257 His(+) colonies were selected on a medium lacking tryptophan, leucine and histidine. The prey fragments of the positive clones were amplified by PCR and sequenced at their 5′ and 3′ junctions. The resulting sequences were used to identify the corresponding interacting proteins in the GenBank database (NCBI) using a fully automated procedure. A confidence score (PBS, for Predicted Biological Score) was attributed to each interaction as previously described (Formstecher et al., 2005). All the predicted interactions are listed in Table S1.

### Generation of endogenously dual-tagged cell lines

For the C-terminal endogenous tagging of LMF1 putative interactors, we introduced a cassette encoding a 3x-Myc tag directly upstream to the stop codon for the gene of interest. This cassette included the selectable marker HXGPRT and was amplified from the vector pLIC-3xmyc-HXGPRT (Huynh and Carruthers, 2009) with primers that included the homology regions of each gene to promote recombination. Insertion of the cassette was facilitated by CRISPR. For this purpose, we replaced the guide RNA in pSAG1-Cas9-GFP-pU6-sgKu80 [modified by (Blakely et al., 2020) from the original pSAG1-Cas9-GFP-UPRT (Shen et al., 2014)] for one targeting the gene of interest (GOI) locus using the Q5 site-directed mutagenesis kit (NEB). Briefly, freshly egressed parasites were harvested and washed once in PBS and resuspended in transfection buffer (Buffer P3, Lonza), and transferred to transfection cuvettes. A total of 2×10^7^ parasites of the LMF1(HA) cell line (Jacobs et al., 2020) were used in each transfection, with 1 µg of the cassette and 1 µg of Cas9 plasmid using the Lonza nucleofection system. Parasites were selected using mycophenolic acid (MPA), and independent clones were collected by serial dilution. All the primers used in this work are listed in Table S2.

### Immunoprecipitation and co-immunoprecipitation assays

To confirm the results of the Y2H screening, we performed co-immunoprecipitations (co-IP) using the LMF1-HA cell line. Intracellular parasites from 10 T175 cultures were released by passing through a 21-gauge needle, spun down (1000 ***g*** for 10 min at 4°C), washed twice in cold PBS, and resuspended in Pierce co-immunoprecipitation lysis buffer (Thermo Fisher Scientific) with protease/phosphatase inhibitor cocktail (100×, Cell Signaling Technology). After 1 h of lysis at 4°C, the samples were sonicated three times for 20 s each time (20% frequency). After sonication, samples were pelleted (11,000 ***g*** for 15 min), and the supernatant was incubated with anti-HA magnetic beads (Thermo Fisher Scientific). Samples were placed in a rocker for 2.5 h before beads were washed once with Pierce co-IP lysis buffer and twice with PBS. Beads were resuspended in 8 M urea and sent for liquid chromatography coupled to tandem mass spectrometry (LC/MS-MS) analysis. Results were narrowed down to proteins that had at least four peptides in the LMF1–HA sample and none in control. To confirm the interaction between LMF1 and its putative interactors, we performed co-immunoprecipitation using the dually tagged cell lines. Intracellular parasites from 2 T175 cultures were syringe-released, and the samples were processed as described for the immunoprecipitation. In the end, the beads and total lysate were resuspended in 2× Laemmli sample buffer (Bio-Rad) supplemented with 5% 2-mercaptoethanol (Sigma-Aldrich) for western blotting.

### Western blotting

Parasite extracts were resuspended in 2× Laemmli sample buffer (Bio-Rad) with 5% 2-mercaptoethanol (Sigma-Aldrich). Samples were boiled for 5 min at 95°C before separation on a gradient 4–20% SDS-PAGE gels (Bio-Rad). Samples were then transferred to nitrocellulose membrane using standard methods for semidry transfer (Bio-Rad). Membranes were probed with rabbit anti-HA (CS29F4 cat. no. 3724S, Cell Signaling Technology), mouse anti-c-Myc (9B11 cat. no. 2276, Cell Signaling Technology) or mouse anti-F_1_B ATPase (made in house) all at a dilution of 1:5000, or rat anti-IMC10 (made in house) at a dilution 1:5000 overnight. Given the high molecular mass of ATPase-guanylyl cyclase, we used 4–20% Tris-acetate SDS gels (Invitrogen) as performed in Yang et al. (2019b). Membranes were then washed and probed with either goat anti-mouse-IgG horseradish peroxidase or goat anti-rabbit-IgG horseradish peroxidase (Sigma-Aldrich) at a dilution of 1:10,000 for 1 h (GE Healthcare). Proteins were detected using SuperSignal West Femto substrate (Thermo Fisher) and imaged using the FluorChem R system (Biotechne). Full original western blots are shown in Fig. S6.

### Duolink^®^ proximity ligation assay

Dually tagged parasites syringe-released from host cells washed twice in cold PBS and fixed with 4% paraformaldehyde for 20 min at room temperature. After fixation, cells were washed once in PBS and then seeded in poly-L-lysine (Sigma-Aldrich) coated glass coverslips. The cells were permeabilized using PBS plus 0.25% Triton X-100 for 30 min at RT. DuoLink^®^ assay (Sigma-Aldrich) was performed according to the manufacturer's instructions with the following modifications: overnight blocking in a humidity chamber and five washes with 1 ml washing buffer per coverslip.

### Generation of the IMC10 iKD strain

To generate the IMC10 inducible knockdown (iKD) strain, we introduced a cassette encoding a transactivator protein (TATi) and a tetracycline responsive element (TRE) upstream of the IMC10 start codon (Salamun et al., 2014). The cassette was amplified from the vector pT8TATi-HXGPRT-tetO7S (Salamun et al., 2014) with primers that included the homology regions corresponding to the upstream region of the *IMC10* gene. The Cas9 guide was made using the pSAG1-Cas9-GFP-pU6-sgKu80 (Blakely et al., 2020) as a template, and the sequences were introduced with the Q5 site-directed mutagenesis kit (NEB). Transfection of both the TATi cassette and the Cas9 and guide RNA vector was performed as above. Correct integration of the TATi insert was validated by PCR. To confirm IMC10 knockdown, freshly lysed parasites were seeded in a confluent HFF monolayer in a T25 flask, with or without 0.5 µg/ml ATc (Sigma Aldrich, cat. no. 94664-10MG). After 24 h, parasites were syringe-released using a 21-gauge needle and washed twice with PBS (1000 ***g*** for 10 min at 4°C). Total RNA was isolated using TRIzol following the manufacturer's instructions. RNA was treated with RNase-Free DNase and quantified by NanoDrop. A total of 500 ng of total RNA was used for cDNA synthesis using a SuperScript® III First-Strand kit (Invitrogen), following the manufacturer's instructions. PCRs were performed using the cDNA to amplify a 150 bp product from IMC10 and tubulin as a control. Primers are listed in Table S2.

### Production of IMC10 antibody

Sequences encoding residues 238–560 of IMC10 were amplified and cloned into the pET28 His6 TEV LIC bacterial expression vector, which includes an N-terminal 6× histidine tag using primers P1-2 (Addgene plasmid #29653; deposited by Scott Gradia). The construct was transformed into BL21(DE3) *Escherichia coli*, and protein expression was induced with 1 mM IPTG. The protein was then purified using Ni-NTA agarose chromatography under denaturing conditions as described previously (Bradley et al., 2005). The purified protein was dialyzed into PBS to remove the urea, and five injections containing ∼500 µg protein were inoculated into rats over the course of 3 months (Cocalico Biologicals). The resulting sera were screened by IFA and western blotting.

### Immunofluorescence assays

For all immunofluorescence assays (IFAs), infected HFF monolayers were fixed with 3.5% paraformaldehyde, quenched with 100 mM glycine, and blocked with PBS containing 3% bovine albumin serum (BSA). Cells were permeabilized in PBS containing 3% BSA and 0.25% Triton X-100 (TX-100). Samples were then incubated with primary antibodies diluted in permeabilization solution for 1 h, washed five times with PBS, and incubated with the respective Alexa Fluor-conjugated antibodies and 5 µg/ml Hoechst 33342 (a nuclear marker; Thermo Fisher Scientific; cat. no. H3570) in PBS for 1 h. The coverslips were washed five times with PBS. After washes, the coverslips were mounted in ProLong Diamond (Thermo Fisher Scientific). Image acquisition and processing were performed using either a Leica DMI6000 B microscope (objective lens HCX PL 100×/1.40-0.7 Oil CS Apochormatic, *z*-step size of 0.3 µm) coupled with LAS X 1.5.1.13187 software, a Nikon Eclipse 80i microscope with NIS-Elements AR 3.0 (objective lens 100×/1.40 Oil Plan Apochromat, *z*-step size of 0.3 µm), or a Zeiss LSM 800 AxioObserver microscope with an AiryScan detector using a ZEN Blue software (version 3.1). The images in this microscope were acquired using a 63× Plan-Apochromat (NA 1.4) objective lens. All images acquired from this microscope were acquired as *Z*-stacks with an *XY* pixel size of 0.035 µm and a *Z*-step size of 0.15 µm. All images then underwent Airyscan processing using ZEN Blue (Version 3.1, Zeiss, Oberkochen, Germany). Images were processed and analyzed using FIJI ImageJ 64 Software. Primary antibodies used in this study are rabbit anti-HA (C29F4 Cell Signaling, 1:1000 dilution), mouse anti-Myc (9B11 Cell Signaling, 1:1000), rabbit anti-acetyl Tubulin (Lys40) (Millipore ABT241, 1:2000), rat anti-IMC3 (1:2000, Marc-Jan Gubbles, Boston College, MA, USA), rabbit anti-IMC6 (1:2000, made in house), mouse anti-F1B ATPase (1:5000, made in house), rabbit anti-ACP (1:5000, Michael Reese, University of Texas Southwestern, TX, USA) and mouse anti-SERCA (1:1000, David Sibley, Washington University School of Medicine, MO, USA). Secondary antibodies included Alexa Fluor 594- or Alexa Fluor 488-conjugated goat anti-rabbit-IgG and goat anti-mouse-IgG (Invitrogen), all used at 1:1000.

### Ultrastructure expansion microscopy

Ultrastructure Expansion Microscopy (U-ExM) was performed as described previously (Liffner and Absalon, 2021) with the following modification: the parasites were seeded in an HFF monolayer grown on glass coverslips in 24-well plates. Primary antibodies used were rabbit anti-HA (C29F4 Cell Signaling, 1:100 dilution), mouse anti-Myc (9B11 Cell Signaling, 1:500), rat anti-IMC3 (made in house, 1:1000) and rabbit AtpB (AS05 805 Agrisera, 1:2500). Secondary antibodies used in this study were Alexa Fluor NHS 405 NHS-ester, Alexa Fluor 647- Alexa Fluor 594- or Alexa Fluor 488-conjugated goat anti-rabbit-IgG and goat anti-mouse-IgG (Invitrogen). Secondary antibodies were used at 1:1000, except for the Alexa Fluor NSH-ester 405, which was used at 1:250. Images were acquired both in the LSM 800 and 900 microscopes. LSM 900 lenses have the same configurations as previously described for the LMS800. Images were AiryScan Processed using the Zen Blue (3.1) software. After the second round of expansion, the gels were measured with a ruler, and the expansion factor (the gel size in relation to the coverslip) was determined. For all measurements based on the U-ExM images, we used an average of 4× expansion. The distances measured were divided by the expansion factor.

### Pellicle extraction

Pellicle extraction was performed as previously described (Gilk et al., 2006) with some modifications. Briefly, 1×10^8^ parasites were resuspended in PBS containing 1% deoxycholate (DOC, v/v). After one cycle of sonication (20% frequency for 20 s on ice), the extract was centrifuged (15,000 ***g*** at 4°C) for 30 min. Part of the extract was recovered for IFA, and the other part was boiled in 2× Laemmli buffer (Bio-Rad) supplemented with 5% 2-mercaptoethanol for western blot analysis.

### Phenotypic characterization of the IMC10 iKD strain

For the plaque assays, 500 freshly egressed parasites were seeded in a confluent HFF monolayer in 12-well plates, with or without 0.5 µg/ml ATc. After 5 days of incubation, cultures were fixed with methanol for 15 min and stained with Crystal Violet. Plaques were imaged using a Protein Simple imager, and the cleared area was calculated using the ColonyArea plugin (Guzmán et al., 2014) on FIJI software. The experiment was performed in five biological replicates, each with three technical replicates. The individual plaque area was calculated using the Viral Plaque macro on FIJI software (Cacciabue et al., 2019). Doubling assays were performed in 24-well plates as described previously (Yang et al., 2019a). The percentage of vacuoles with a particular number of parasites (i.e. 1, 2, 4, 8, 16, 32 and 64, and including an abnormal number of parasites) was tabulated for all strains, and conditions were monitored at 48 h after infection. The experiment was performed in triplicate, each one containing three technical replicates.

For the mitochondrial morphology counts, HFFs infected with the iKD strain were grown with or without 0.5 µg/ml ATc for 24 and 48 h. IFA was performed as described above using mouse anti-F_1_B ATPase and anti-rat IMC3. Samples were examined by a researcher who was blind to the experimental conditions, and at least 150 non-dividing vacuoles with an intact IMC were inspected. Three mitochondrial morphological categories were quantitated: lasso, sperm-like and collapsed (Jacobs et al., 2020; Ovciarikova et al., 2017). The other mitochondrial phenotypes accessed during the image analysis were counted and categorized as: broken lasso (Mallo et al., 2021), amitochondriate parasites and extracellular mitochondrial material (Jacobs et al., 2020). The division phenotypes were quantified using the same images by counting the number of synchronous and asynchronous vacuoles and the number of daughter cells in each dividing parasite. Synchronous vacuoles are those in which all parasites are in the same stage of division. At least 150 vacuoles were counted per condition. Experiments were performed in biological triplicates. For all mitochondrion-related phenotypes, the images were composed of 35–40 *z*-steps, *z*-stacked and deconvolved.

To calculate the number of mitochondrion–IMC contact sites per cell, we treated the parasites (or not) with ATc for 48 h. We inspected 50 parasites per condition and quantified the number of visual contacts (points where the mitochondrion touches the IMC) in 50 cells per condition. Experiments were performed in triplicate. To calculate the distance from the mitochondrion to the pellicle in the expanded parasites, we inspected 30 parasites per condition (with and without ATc) in biological duplicates. Parasites were stained for IMC using the IMC3 antibody, and mitochondrion was detected using the NHS-ester staining as a reference. The distance was measured based on the closest point between both organelles. It was measured as a single contact in each individual cell. Images were processed and analyzed using FIJI ImageJ 64 Software. The total distance in expanded parasites was then converted into ‘corrected distance’ by modifying to the expansion factor of the gel (4×).

### Transmission electron microscopy

HFFs monolayers were infected with iKD-IMC10 parasites for 24 h in the presence (or not) of ATc. The cultures were washed 3× in PBS and fixed with 2% glutaraldehyde and 2% paraformaldehyde in 0.1 M sodium phosphate buffer (pH 7.4) for 1 h. After fixation, cells were harvested by scraping the monolayer and centrifugation at 1000 ***g*** for 5 min. Cells were post-fixed for 95 min in the dark in 1% osmium tetroxide (OsO_4_) diluted in ultrapure water. After fixation, the cells were dehydrated in increasing concentrations of ethanol (50–100%) at room temperature and embedded in EPON resin (Electron Microscopy Sciences). Ultrathin sections (70–80 nm) were obtained in a UCT Ultracut with FCS (Leica), and the sections were visualized in a Tecnai Spirit OR (Thermo Fisher Scientific) equipped with AMT CCD Camera (Advanced Microscopy Techniques). Images were acquired at the Electron Microscopy Core Facility at the Indiana University School of Medicine.

### Statistical analyses

Data were analyzed using GraphPad Prism software (version 9.00, La Jolla, CA). Analyses were performed using either unpaired, one-tailed Student's *t*-test or one-way ANOVA with Tukey post-correction.

## Supplementary Material

10.1242/joces.260083_sup1 Supplementary information
