# Supplementary material for: IMC10 and LMF1 mediate mitochondrial morphology through mitochondrion–pellicle contact sites in Toxoplasma gondii
Source: J Cell Sci. 2022 Nov 15;135(22):jcs260083. doi: 10.1242/jcs.260083 (PMC9845740; doi:10.1242/jcs.260083)
Supplement: Supplementary information [file joces-135-260083-s1.pdf]

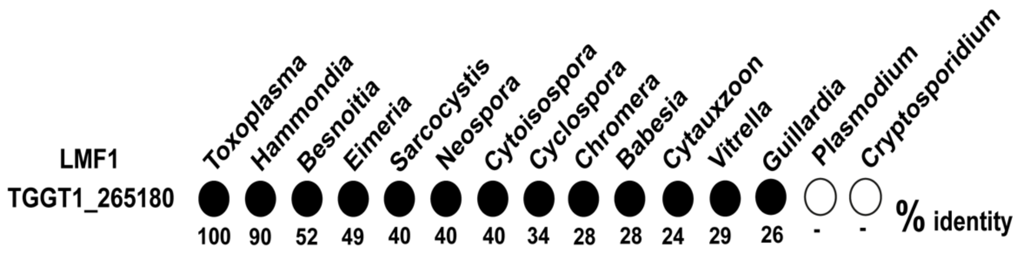

Fig. S1. Domains and phylogenetic analysis of LMF1. List of apicomplexan parasites with LMF1 orthologs. Black filled circle means the presence of an orthologue, and a white-filled circle means the absence of an LMF1 orthologue. Bellow the circles, the percentage of amino acid identity of each homologue found by reciprocal BLASTp.

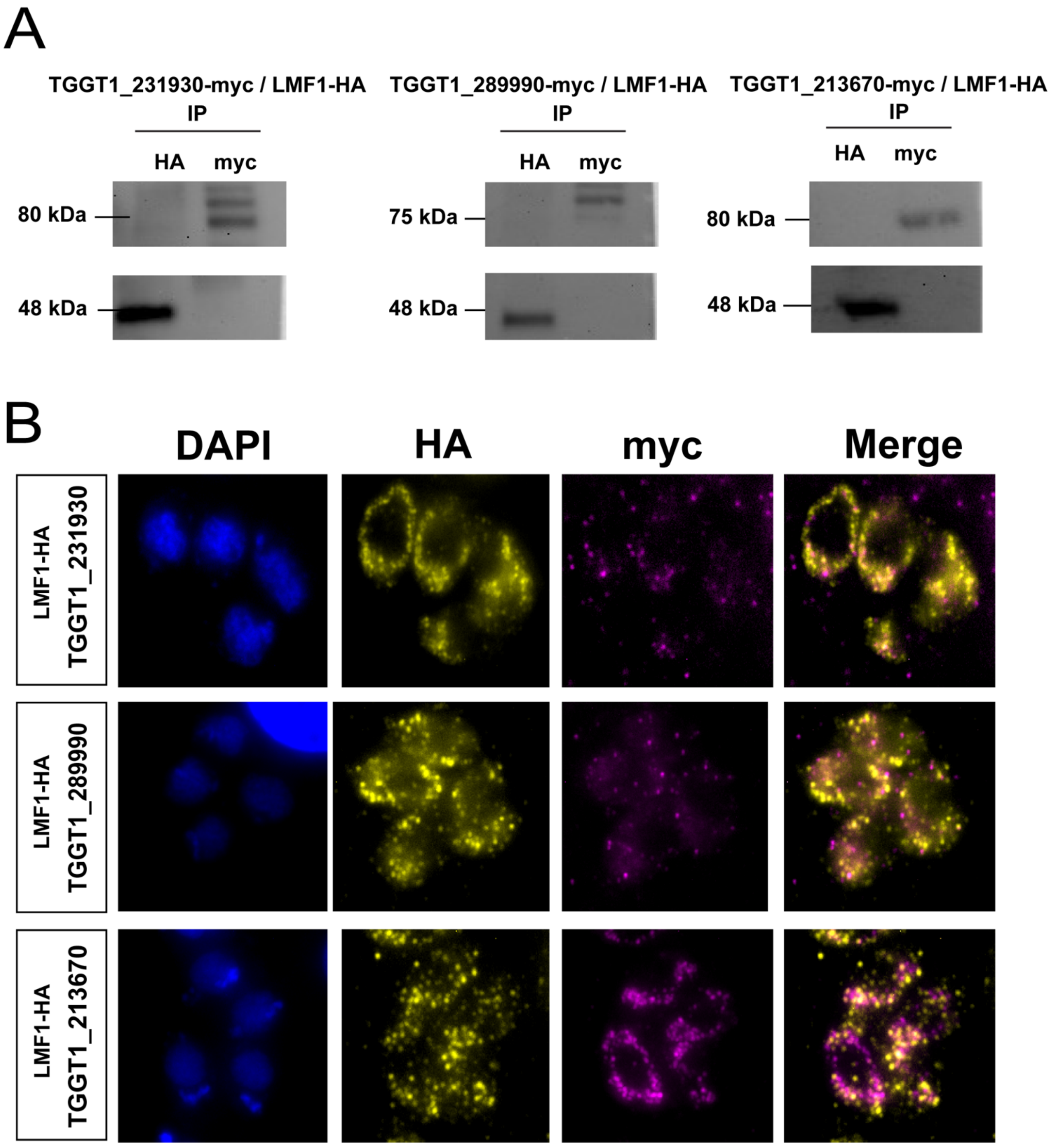

Fig. S2. Characterization of LMF1 interactors. To investigate the localization of LMF1 interactors, we introduced sequences encoding an N-terminal Myc tag to the endogenous locus in the parasite strain expressing an HA-tagged LMF1. A) Reciprocal co-immunoprecipitation of putative LMF1 interactors was performed for the strains expressing LMF1-HA and either TGGT1_231930-Myc, TGGT1_289990-Myc, or

Journal of Cell Science • Supplementary information

TGGT1_213670-Myc. For each of the three dually tagged parasite strains, proteins were immunoprecipitated with either anti-HA or anti-Myc conjugated beads and probed with either Myc (for the interactor) and for HA (for LMF1). B) Intracellular parasites expressing the Myc tagged versions of TGGT1_231930-myc, TGGT1_289990-myc and TGGT1_213670-myc were stained for HA (yellow) and myc (magenta).

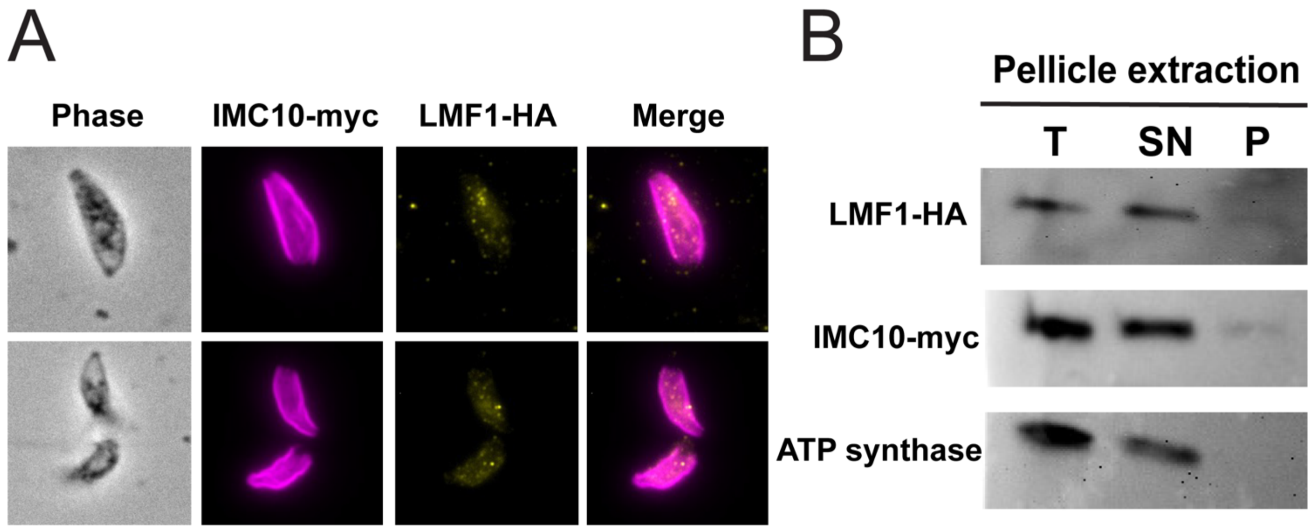

Fig S3. Localization of LMF1 in isolated pellicles. Pellicles were extracted from intracellular parasites with deoxycholate (DOC) and used for IFA (A) and Western Blots (B). A) IFA of DOC extracted pellicles showing IMC10 (magenta) and LMF1 (yellow). B) Representative western blots from total parasite extract (T) and both the supernatant (SN) and pellet (P) from the DOC extracted fraction. Western blots were probed for LMF1 (anti- HA), IMC10 (anti-Myc), and the ATP synthase subunit B. Only IMC10 and LMF1 are detected in the pellet fraction, which contains the parasite pellicle.

Journal of Cell Science • Supplementary information

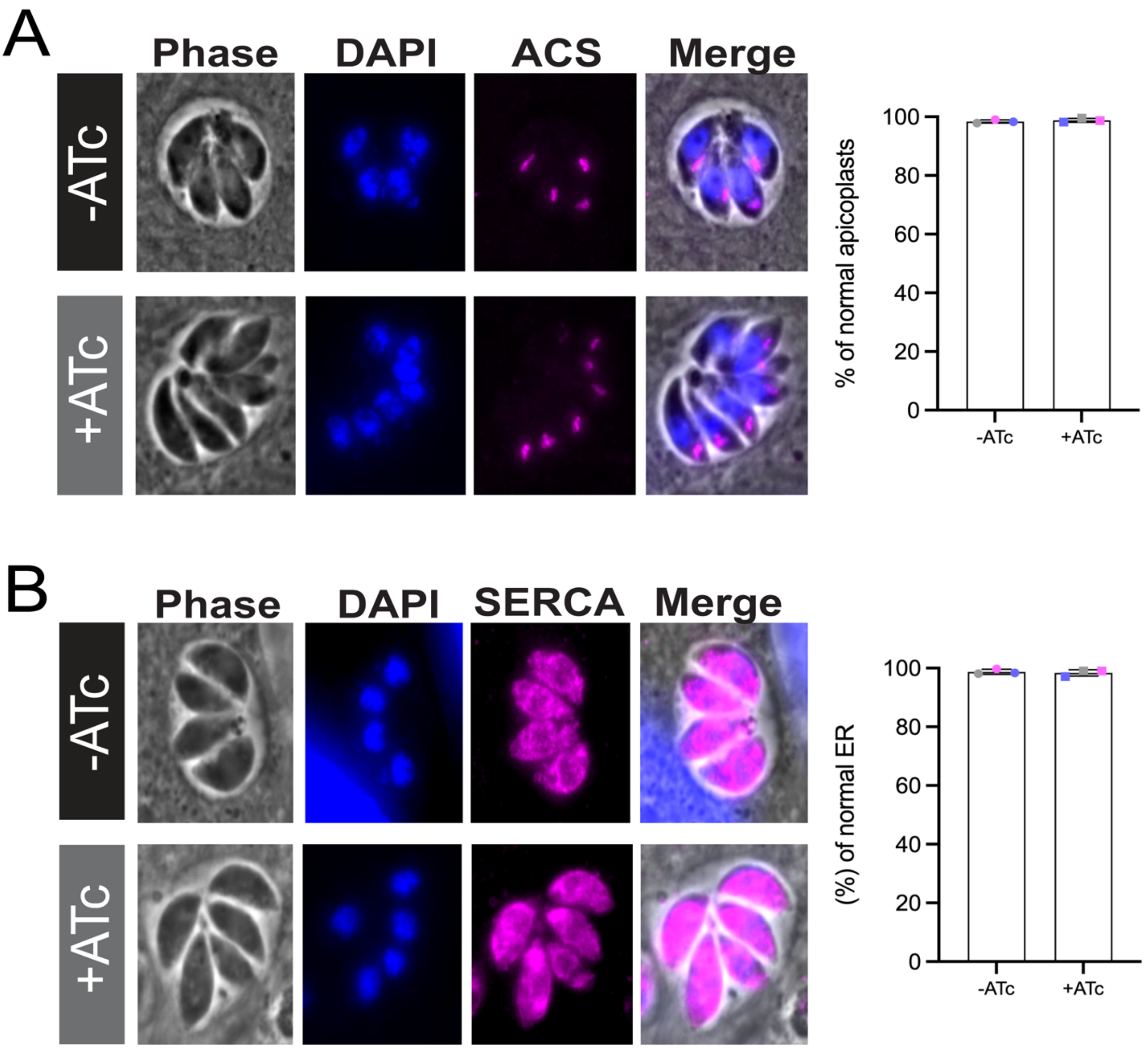

Fig. S4. IMC10 knockdown does not affect apicoplast or endoplasmic reticulum (ER) morphology. IFA of parasites stained for DAPI (blue), apicoplast, and ER (magenta) showing the shape of both structures in parasites maintained with and without ATC for 48h. A) Apicoplast morphology at 48h. B) ER morphology at 48h. All graphs represent the percentage of vacuoles with the related phenotype. At least 150 vacuoles per sample were inspected. For all graphs, n = 3. Error bars represent standard deviation.

Journal of Cell Science • Supplementary information

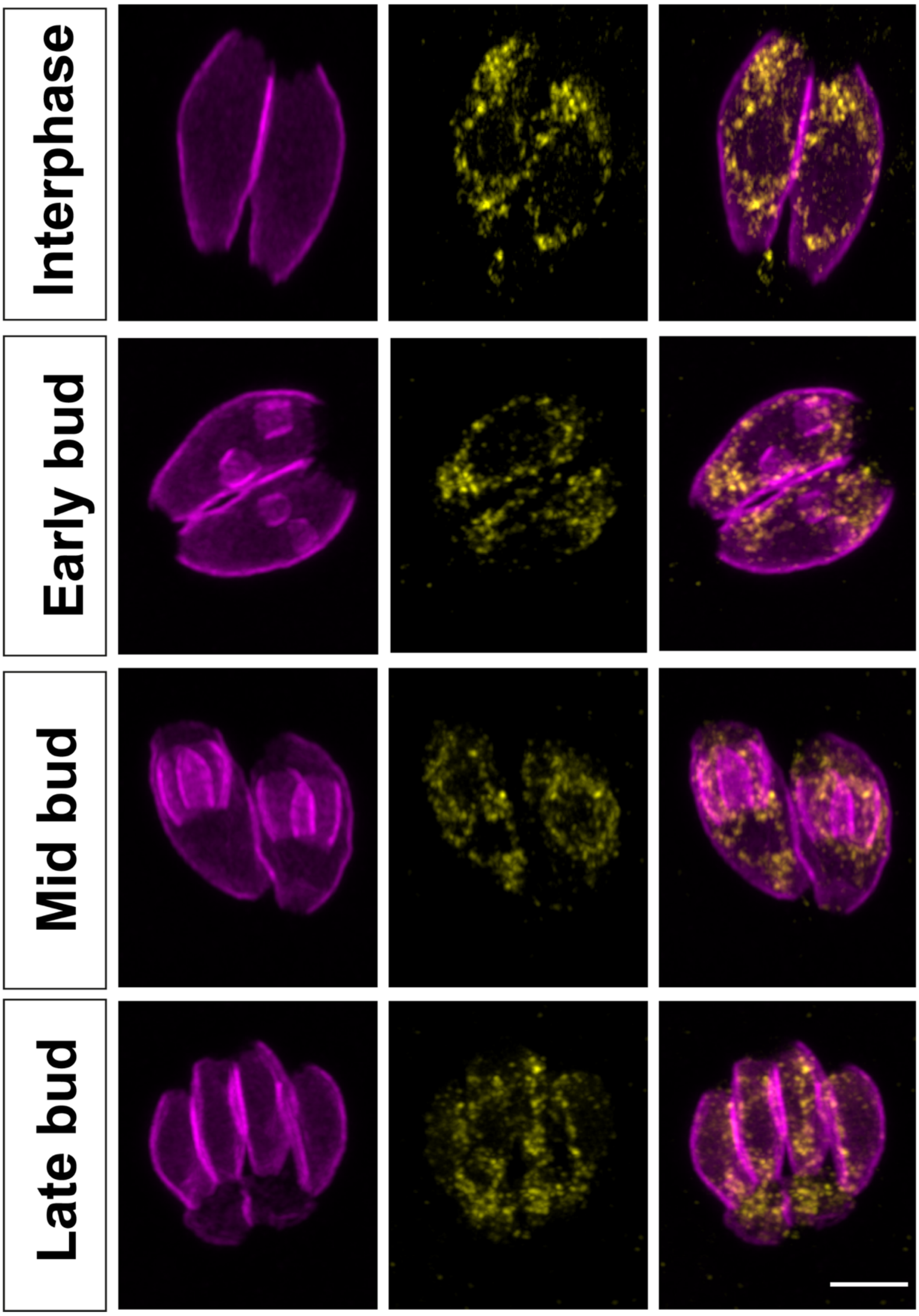

Fig. S5. LMF1 and IMC10 interact during mitochondrial distribution. IFA of intracellular parasites stained for LMF1-HA (yellow) and IMC10-Myc (magenta). Scale bar = 5µm. Images were acquired using a Zeiss LSM 800 microscope with Airyscan processing.

Western blots Figure 1B

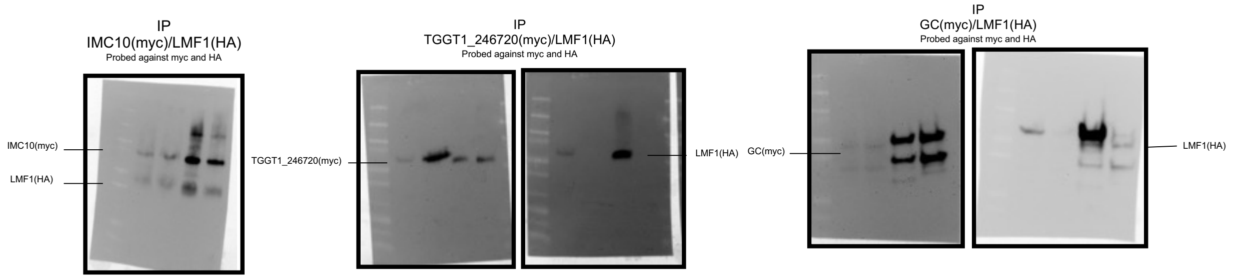

Western blots Figure S2A

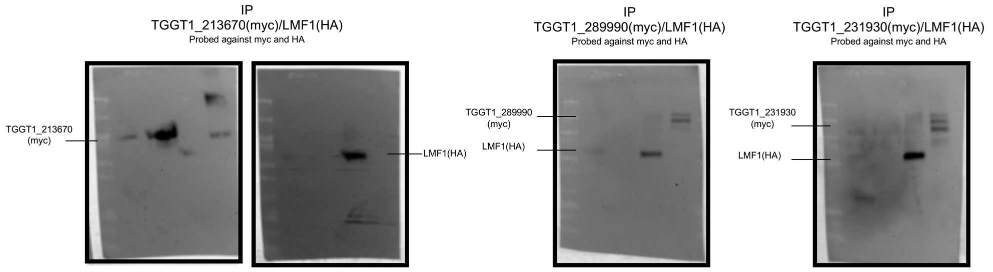

Western blots Figure 3C and D

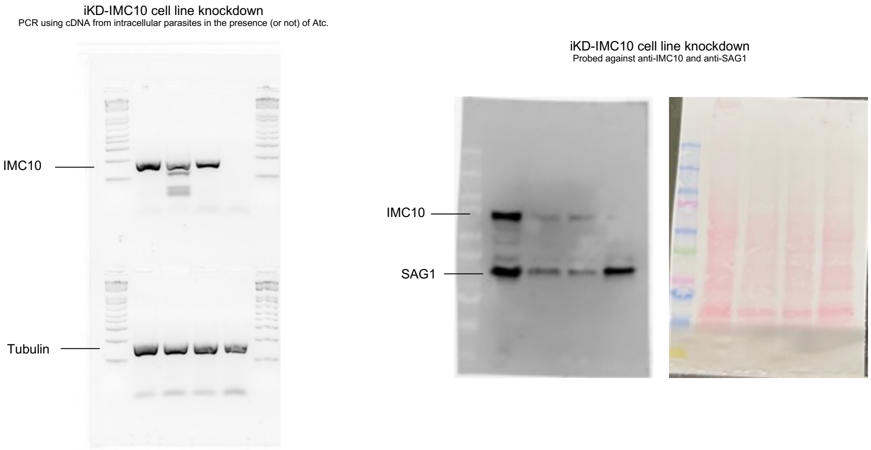

Western blots Supplemental Figure S3

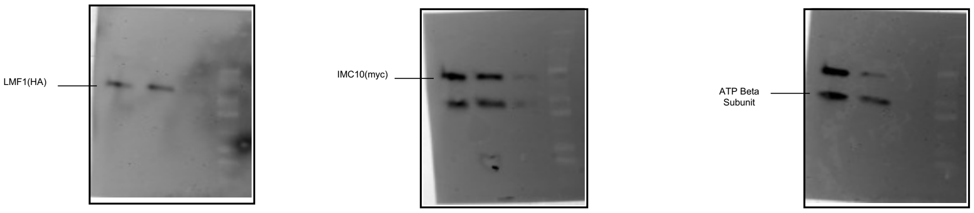

Fig. S6. Original images for the western blots showed in this work with molecular weight.

Journal of Cell Science • Supplementary information

Table S1. List of candidates LMF1 interactors. Listed are proteins identified by the yeast two-hybrid (Y2H) screen. Included are the Gene ID, the gene annotation, and the global PrBS score for the likelihood of interaction in Y2H.

Gene Name (Best Match)	GenBank ID (NCBI)	ToxoDB ID	Global PBS
**ALV12**	XM_002367901.1	TGME49_230210	A
**SRS20A**	XM_002369184.2	TGME49_285870	A
**elongation factor 1-gamma, putative**	XM_002371696.2	TGME49_300140	A
**tRNA ligase class II core domain (G, H, P, S and T) domain-containing protein**	XM_002370233.2	TGME49_295050	A
**MIC2**	XM_018779175.1	TGME49_201780	B
**Sel1 repeat-containing protein**	XM_018780375.1	TGME49_233810	B
**guanylyl cyclase**	XM_018781054.1	TGME49_254370	B
**hypothetical protein**	XM_002366901.2	TGME49_244530	B
**myosin A**	XM_002368901.2	TGME49_235470	B
**phosphoglycerate mutase family protein**	XM_018781182.1	TGME49_258540	B
**clathrin heavy chain, putative**	XM_002368438.2	TGME49_290950	C
**hypothetical protein**	XM_002365609.1	TGME49_269690	C
**hypothetical protein**	XM_002366715.2	TGME49_242260	C
**hypothetical protein**	XM_002364969.2	TGME49_257760	C
**hypothetical protein**	XM_018779329.1	TGME49_205360	C
**hypothetical protein**	XM_002371294.2	TGME49_217680	C
**hypothetical protein**	XM_002370465.2	TGME49_278030	C
**hypothetical protein**	XM_018782763.1	TGME49_313380	C
**ACC2**	XM_002370599.2	TGME49_218560	D
**CDPK7**	XM_018780218.1	TGME49_228750	D
**ClpB, putative**	XM_018781733.1	TGME49_275690	D
**DEAD-family helicase**	XM_002371178.2	TGME49_298020	D
**DEAD/DEAH box helicase domain-containing protein**	XM_002370173.1	TGME49_294350	D
**DNA polymerase family B protein**	XM_018782969.1	TGME49_319860	D
**FRM1**	XM_018779357.1	TGME49_206430	D
**GRA5**	XM_002369230.2	TGME49_286450	D
**GRA6**	XM_002371898.2	TGME49_275440	D
**LisH protein**	XM_002364639.2	TGME49_314900	D
**PCI domain-containing protein**	XM_002366378.2	TGME49_227960	D
**RNA pseudouridine synthase superfamily protein**	XM_002370387.2	TGME49_306660	D
**Sec7 domain-containing protein**	XM_018781442.1	TGME49_266830	D
**WD domain, G-beta repeat-containing protein**	XM_018781644.1	TGME49_272040	D
**XPA binding protein 2 family protein**	XM_002370276.2	GME49_305240	D
**crooked neck family 1 protein isoform 2, putative**	XM_018781534.1	TGME49_269200	D
**eukaryotic initiation factor-3, subunit 8, putative**	XM_018782235.1	TGME49_294620	D
**histidine acid phosphatase superfamily protein**	XM_018779286.1	TGME49_204080	D
**hypothetical protein**	XM_002366120.2	TGME49_224870	D
**hypothetical protein**	XM_002367018.2	TGME49_246720	D
**hypothetical protein**	XM_002366895.2	TGME49_244470	D

Journal of Cell Science • Supplementary information

**hypothetical protein**	XM_018780307.1	TGME49_231930	D
**hypothetical protein**	XM_018782084.1	TGME49_289520	D
**hypothetical protein**	XM_002368014.1	TGME49_231840	D
**hypothetical protein**	XM_018779183.1	TGME49_202040	D
**hypothetical protein**	XM_018780814.1	TGME49_247290	D
**hypothetical protein**	XM_018781060.1	TGME49_254470	D
**hypothetical protein**	XM_018781216.1	TGME49_259720	D
**hypothetical protein**	XM_018780111.1	TGME49_225745	D
**hypothetical protein**	XM_018779615.1	TGME49_213670	D
**hypothetical protein**	XM_018781450.1	TGME49_267020	D
**hypothetical protein**	XM_018781196.1	TGME49_258870	D
**hypothetical protein**	XM_018780237.1	TGME49_229790	D
**hypothetical protein**	XM_018780016.1	TGME49_223760	D
**hypothetical protein**	XM_002370104.2	TGME49_293430	D
**hypothetical protein**	XM_018782540.1	TGME49_308070	D
**hypothetical protein**	XM_018781162.1	TGME49_258070	D
**hypothetical protein**	XM_002367593.2	TGME49_203520	D
**hypothetical protein**	XM_002369737.1	TGME49_318390	D
**hypothetical protein**	XM_018781098.1	TGME49_255300	D
**hypothetical protein**	XM_002367173.2	TGME49_248680	D
**hypothetical protein**	XM_002369325.2	TGME49_252880	D
**hypothetical protein**	XM_018781674.1	TGME49_272695	D
**hypothetical protein**	XM_002368383.2	TGME49_289990	D
**kinesin heavy chain, putative**	XM_002365916.1	TGME49_273560	D
**myosin H**	XM_002366784.2	TGME49_243250	D
**root hair defective 3 gtp-binding protein (rhd3) protein**	XM_018782846.1	TGME49_314970	D
**tetratricopeptide repeat domain containing protein**	XM_002369289.2	TGME49_252220	D
**tetratricopeptide repeat-containing protein**	XM_002371471.2	TGME49_295730	D
**ubiquitin family protein**	XM_002371852.2	TGME49_304680	D
**zinc finger (CCCH type) motif-containing protein**	XM_018780934.1	TGME49_250690	D
**hypothetical protein**	XM_002370830.2	TGME49_215520	E

Journal of Cell Science • Supplementary information

Table S2. LMF1 interactors identified by immunoprecipitation. Listed are proteins that had at least four peptides in the LMF1 IP and zero in control (parental parasites). LMF1 is in yellow and IMC10 in pink. Proteins that are known to localize to the pellicle are highlighted in grey. Included are the Gene ID, the gene annotation, and the number of peptides detected by mass spectrometry.

Gene ID	**Product Description**	Total Peptides
**TGGT1_265180**	**LMF1**	**53**
TGGT1_286580	IMC17	40
TGGT1_219320	GAP50	38
**TGGT1_230210**	**IMC10**	**30**
TGGT1_324600	HSP20	26
TGGT1_222220	IMC7	23
TGGT1_308860	AC3	21
TGGT1_232410	TrxL1	20
TGGT1_258410	PhIL1	16
TGGT1_248700	IMC12	13
TGGT1_241170	hypothetical	12
TGGT1_252360	ROP24	10
TGGT1_219270	GAPM2a	9
TGGT1_219310	DnaK family protein (HSP70)	9
TGGT1_249970	Acylated Pleckstrin-Homology (APH)	7
TGGT1_262050	ROP39	6
TGGT1_287500	T complex chaperonin, putative	4
TGGT1_316540	ISP3	4

Journal of Cell Science • Supplementary information

Table S3. Sequences of primers were used in this study. Small caps indicate overhangs with homology to the targeted region for recombination.

Use	#	Primers (5'-3')
IMC10 guide RNA targeting	P001	acaacacacaGTTTTAGAGCTAGAAATAGC
	P002	gggacgtctcAACTTGACATCCCCATTTAC
IMC10 C-terminal homology regions for myc tagging	P003	GCCAGCGGAGTAGGATTGGGCGAAGAGGCACAGATCAGCGCCttaattaaaattggaagtggagg
	P004	CATGCCCTGTCCCTAAAAATTAGTTCCCTTTCTCAGTTGTAGGTTTTCCCAGTCACGACG
TGGT1_246720 guide RNA	P005	tggatgtcagGTTTTAGAGCTAGAAATAGC
	P006	aacctctttcAACTTGACATCCCCATTTAC
TGGT1_246720 homology regions for myc tagging	P007	TACCACGGCCACGGAAATATCATCACATGGGGAAAATCTTGTttaattaaaattggaagtggagg
	P008	ATCACGCGTCCGGCTGGGTTTCTTTTAACGGAGACATACGAAGTTTTCCCAGTCACGACG
ATPase-GC guide RNA	P009	aacgcagaacAACTTGACATCCCCATTTAC
	P010	gctccagaggGTTTTAGAGCTAGAAATAGC
ATPase-GC homology regions for Myc tagging	P011	CCTTCGGATATAGGGTCGACACCTGGCTCTGCACTCGGGTCGttaattaaaattggaagtggagg
	P012	GCCTCTGTCTGTCTCGACTGCCCGAAGCGGCAGGACACAGACGTTTTCCCAGTCACGACG
TGGT1_213670 guide RNA	P013	ggtgtgttgcGTTTTAGAGCTAGAAATAGC
	P014	cacccccgtcAACTTGACATCCCCATTTAC
TGGT1_213670 homology regions for myc tagging	P015	GCATGCATCAATGCGGATCTGCTTGCCGGTGCTAAATCCCCAttaattaaaattggaagtggagg
	P016	TCTTCAAGTCTAGGTCGGCGAATAGTTCGTACCACCTAGCATGTTTTCCCAGTCACGACG
Guide RNA change using Q5	P017	gcgagggacgGTTTTAGAGCTAGAAATAGC
	P018	actctgttgcAACTTGACATCCCCATTTAC
TGGT1_246720 homology regions for Myc tagging	P019	CGAGACGAGGAAGACGACGTTTCCTCGCTTTCCGAAGACGAGttaattaaaattggaagtggagg
	P020	GAACAGAAGACTCCCGTGCACAGCGTCTGCTTCCATCGTGTGTGTTTTCCCAGTCACGACG
Guide RNA change using Q5	P021	gagacgaattGTTTTAGAGCTAGAAATAGC
	P022	aggtggagtcAACTTGACATCCCCATTTAC
TGGT1_246720 homology regions for Myc tagging	P023	CTCCGACAGCGGCCTGCAGCAGGATTCTCTCTGCAGAACGAGttaattaaaattggaagtggagg
	P024	GGAGACAAGGAGCCCTTGGTACTTTAGTGCCACGCACGCTCAGTTTTCCCAGTCACGACG
Guide RNA change using Q5	P025	taataggagtGTTTTAGAGCTAGAAATAGC
	P026	tcagaggtccAACTTGACATCCCCATTTAC
	P027	TTCAGGAATCGCCAAACAAACATTCCAGTGGCTTTTCCTACTcatgtttgcggatccgggg
	P028	TGTGTGTGTGCGAAAAGGGAATCGAGGATAGCGTTAGGACGACAGGTCCTCCTCGGAGATGA
qPCR IMC10	P029	AGGAGGTCGAGGTTGTTCAG
	P030	TCAAAGGAGAGCAGTAGGGC
qPCR Tubulin	P031	TGTCGCTGAGATCACCAACT

Journal of Cell Science • Supplementary information

	P032	AGTCGACGAACTGGATGGTT
Promoter replacement confirmation	P033	gcctacgtgacttgctgatg
	P034	Gagtggaggattcgcagtct
Generation of pET-LIC-TEV IMC10 expressing construct	P035	tacttccaatccaatgcaGGAACTGCCACACACAAGGT
	P036	ttatccacttccaatgttattaGGCGCTGATCTGTGCCTCTT

Journal of Cell Science • Supplementary information

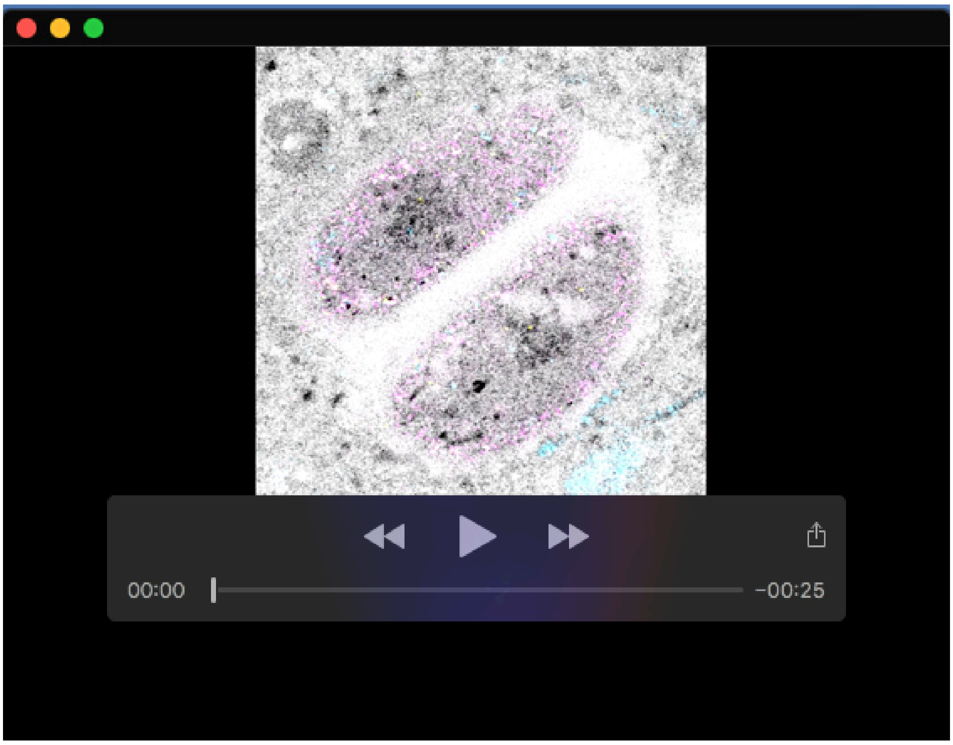

Movie 1. Full Z-stacking of expanded parasites showed in Fig 1B. Images were acquired using a Zeiss LSM 900 with Airyscan processing. The movie was edited and processed in FIJI. The IMC is represented in magenta, LMF1 in yellow and Mitochondrion in cyan.

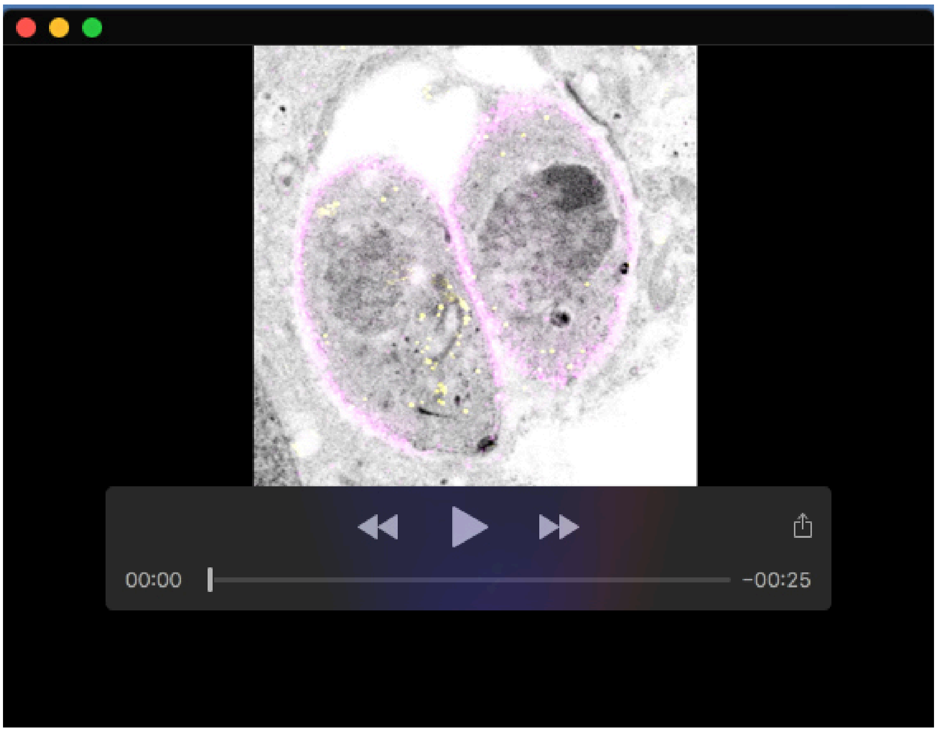

Movie 2. Full Z-stacking of expanded parasites during interphase as shown in Fig 7A. Images were acquired using a Zeiss LSM 900 with Airyscan processing. The movie was edited and processed in FIJI. The IMC is represented in magenta, LMF1 in yellow.

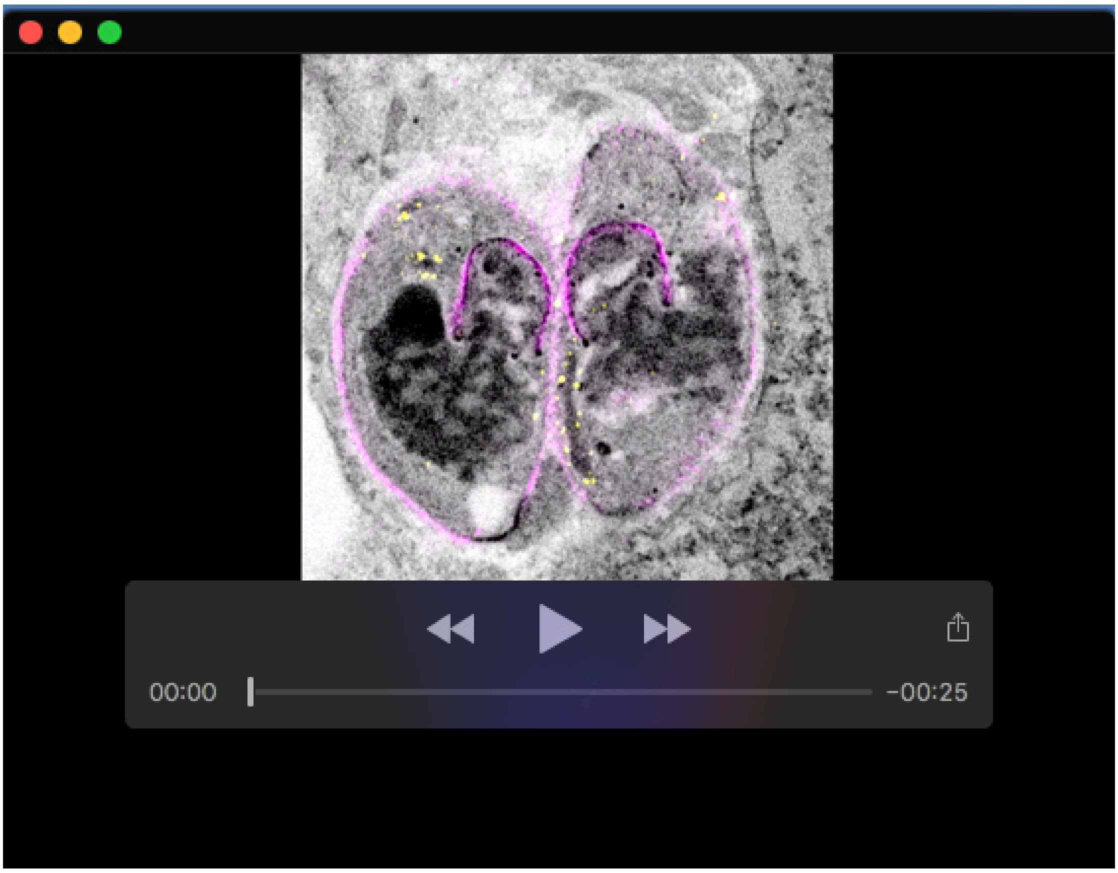

Movie 3. Full Z-stacking of expanded parasites undergoing division during an early stage as shown in Fig 7A. Images were acquired using a Zeiss LSM 900 with Airyscan processing. The movie was edited and processed in FIJI. The IMC is represented in magenta, LMF1 in yellow.

Journal of Cell Science • Supplementary information

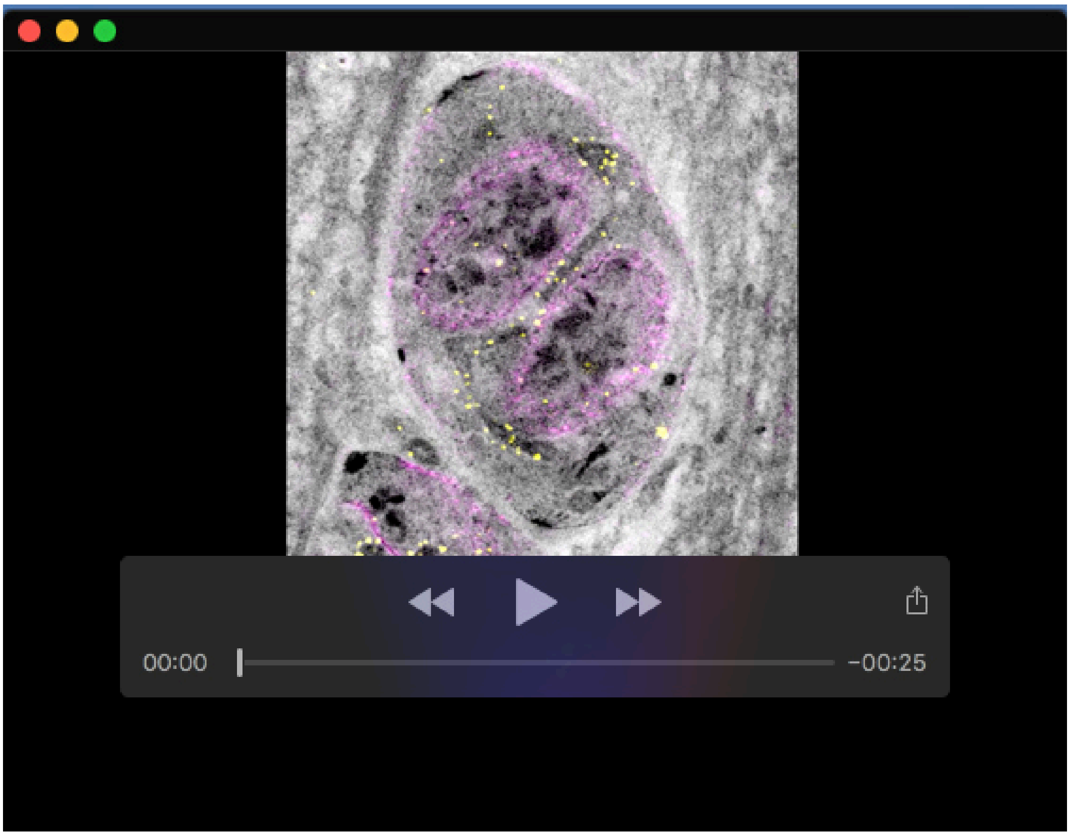

Movie 4. Full Z-stacking of expanded parasites undergoing division during a mid-bud stage as shown in Fig 7A. Images were acquired using a Zeiss LSM 900 with Airyscan processing. The movie was edited and processed in FIJI. The IMC is represented in magenta, LMF1 in yellow.

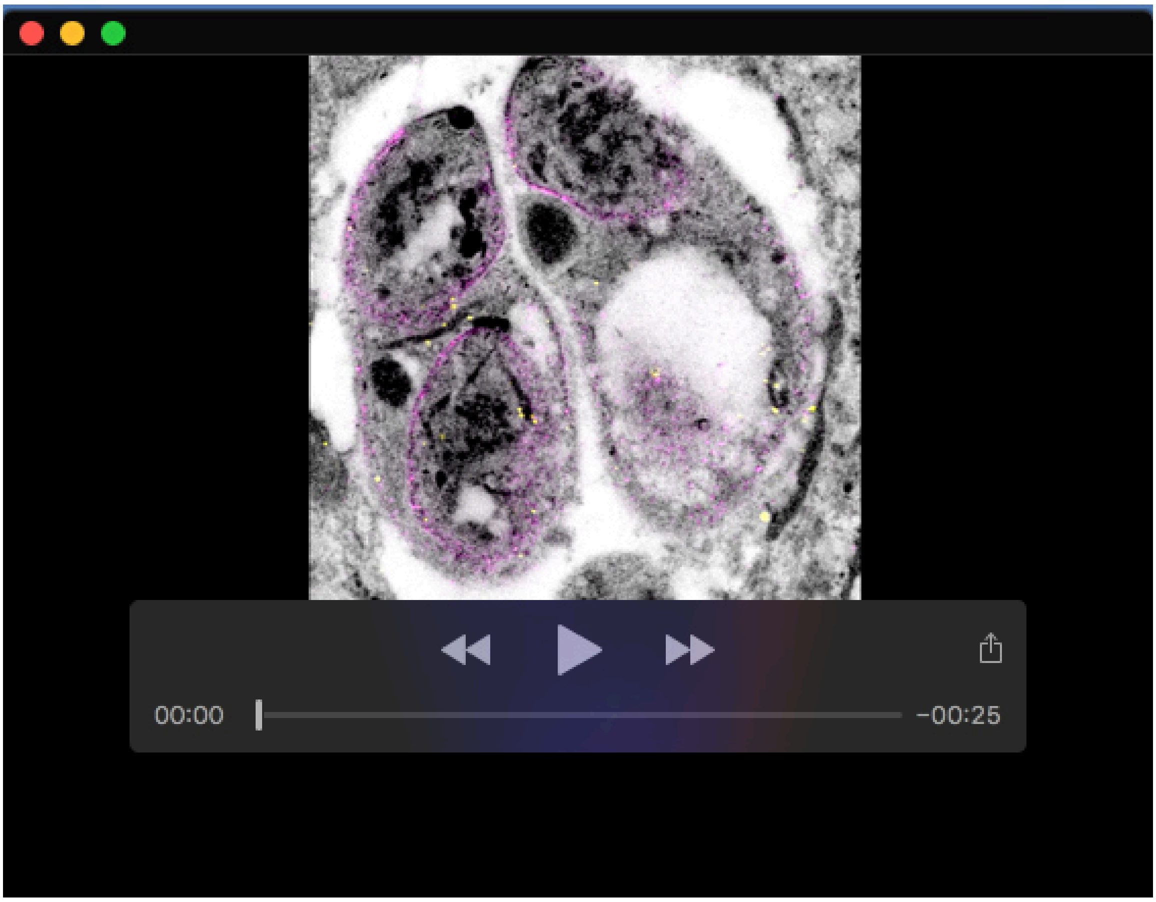

Movie 5. Full Z-stacking of expanded parasites undergoing division during a late stage as 96 shown in Fig 7A. Images were acquired using a Zeiss LSM 900 with Airyscan processing. The movie was edited and processed in FIJI. The IMC is represented in magenta, LMF1 in yellow.

Journal of Cell Science • Supplementary information
